# Real Time Tracking of Nanoconfined Water‐Assisted Ion Transfer in Functionalized Graphene Derivatives Supercapacitor Electrodes

**DOI:** 10.1002/advs.202307583

**Published:** 2024-08-06

**Authors:** Akshay Kumar K. Padinjareveetil, Martin Pykal, Aristides Bakandritsos, Radek Zbořil, Michal Otyepka, Martin Pumera

**Affiliations:** ^1^ Future Energy and Innovation Laboratory Central European Institute of Technology Brno University of Technology Purkyňova 123 Brno 61200 Czech Republic; ^2^ Regional Centre of Advanced Technologies and Materials Czech Advanced Technology and Research Institute (CATRIN) Palacký University Olomouc Olomouc 783 71 Czech Republic; ^3^ Nanotechnology Centre Centre for Energy and Environmental Technologies VŠB–Technical University of Ostrava 17. listopadu 2172/15 Ostrava‐Poruba 708 00 Czech Republic; ^4^ IT4Innovations VŠB–Technical University of Ostrava 17. listopadu 2172/15 Ostrava‐Poruba 708 00 Czech Republic; ^5^ Advanced Nanorobots & Multiscale Robotics Laboratory Faculty of Electrical Engineering and Computer Science VSB – Technical University of Ostrava 17. listopadu 2172/15 Ostrava 708 00 Czech Republic; ^6^ Department of Chemical and Biomolecular Engineering Yonsei University 50 Yonsei‐ro, Seodaemun‐gu Seoul 03722 South Korea; ^7^ Department of Medical Research China Medical University Hospital China Medical University No. 91 Hsueh‐Shih Road Taichung 40402 Taiwan

**Keywords:** confined water molecules, covalent functionalization, energy storage, EQCM, Graphene derivatives

## Abstract

Water molecules confined in nanoscale spaces of 2D graphene layers have fascinated researchers worldwide for the past several years, especially in the context of energy storage applications. The water molecules exchanged along with ions during the electrochemical process can aid in wetting and stabilizing the layered materials resulting in an anomalous enhancement in the performance of supercapacitor electrodes. Engineering of 2D carbon electrode materials with various functionalities (oxygen (─O), fluorine (─F), nitrile (─C≡N), carboxylic (─COOH), carbonyl (─C═O), nitrogen (─N)) can alter the ion/water organization in graphene derivatives, and eventually their inherent ion storage ability. Thus, in the current study, a comparative set of functionalized graphene derivatives—fluorine‐doped cyanographene (G–F–CN), cyanographene (G–CN), graphene acid (G–COOH), oxidized graphene acid (G‐COOH (O)) and nitrogen superdoped graphene (G–N) is systematically evaluated toward charge storage in various aqueous‐based electrolyte systems. Differences in functionalization on graphene derivatives influence the electrochemical properties, and the real‐time mass exchange during the electrochemical process is monitored by electrochemical quartz crystal microbalance (EQCM). Electrogravimetric assessment revealed that oxidized 2D acid derivatives (G–COOH (O)) are shown to exhibit high ion storage performance along with maximum water transfer during the electrochemical process. The complex understanding of the processes gained during supercapacitor electrode charging in aqueous electrolytes paves the way toward the rational utilization of graphene derivatives in forefront energy storage applications.

## Graphical abstract

Covalent functionalization and doping of graphene surfaces —featuring groups such as oxygen, cyano‐, carbon‐fluorine, carboxyl groups, and nitrogen heteroatoms— significantly affects water‐assisted ion transfer as monitored with electrochemical quartz crystal microbalance, modulating the performance of supercapacitor electrodes. Such studies are crucial for advancing energy storage applications with a broader impact across electrochemistry‐related technological domains.

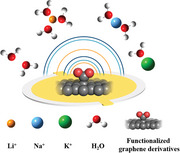

## 1 Introduction

The limited availability of non‐renewable fossil fuel resources along with the massive rise in human population has raised concerns among the global community in combating the growing energy demands. Therefore, the quest for alternative clean energy sources has taken center stage in global research today.^[^ ^1^, ^2^, ^3^, ^4^ ^]^ Fabricating devices for energy storage applications has advanced enormously in the recent past, wherein the key aspect relies on storing electricity efficiently in the form of chemical energy and delivering upon demand for diverse applications.^[^ ^5^, ^6^, ^7^, ^8^ ^]^ From time‐to‐time, advancement in this domain has resulted in several important findings, yet studies focusing on devising an ideal electrode material and devices that are cost‐effective, flexible, efficient, and sustainable, are still in progress. With graphite anode in lithium‐ion batteries still playing a major role in the market,^[^ ^9^ ^]^ studies have expanded in exploring the possibilities and potential of carbon materials, such as 2D graphene^[^ ^10^, ^11^, ^12^ ^]^ and its derivatives,^[^ ^13^, ^14^, ^15^ ^]^ as active electrode material^[^ ^16^, ^17^, ^18^, ^19^, ^20^ ^]^ for electrochemical applications and beyond. Multiple studies are also reported on the influence of functional groups on charge storage,^[^ ^21^, ^22^ ^]^ and on the assessment of ion and/or solvent exchange in activated carbon,^[^ ^23^ ^]^ graphene,^[^ ^24^ ^]^ MXene,^[^ ^25^, ^26^ ^]^ layered double hydroxides^[^ ^27^ ^]^ etc, for the fabrication of efficient energy storage devices.

Since the discovery of graphene in 2004 — along with several other domains — the field of electrochemical energy storage devices has undergone enormous growth.^[^ ^28^, ^29^, ^30^ ^]^ This class of material has gained immense attention in the field of energy storage applications owing to their abundance, low cost, high conductivity, chemical stability, large specific surface area, etc. Ideal fabrication approaches along with optimized and selective synthesis methods are often considered to be the backbone of procuring superior graphene derivatives, especially for the fabrication of active electrode materials for energy storage applications. Covalent/ non‐covalent functionalization,^[^ ^31^, ^32^ ^]^ functionalization with nanoparticles,^[^ ^31^, ^33^ ^]^ doping/heteroatom functionalization^[^ ^34^, ^35^, ^36^ ^]^ are certainly well‐known approaches toward the modification of graphene that aid in enhancing their properties for designing application‐specific electrode materials and devices. However, the low reactivity of graphene^[^ ^37^, ^38^ ^]^ and the required harsh reaction conditions inhibit direct covalent functionalization of graphene, calling for selective and improved experimental procedures. Graphene oxide (GO) can be considered as one such example, whereby the harsh preparation conditions result in various oxygen functionalities such as epoxide, carboxylic, carbonyl groups, etc.^[^ ^39^, ^40^ ^]^ Thus, such dense functionalization under uncontrolled conditions often renders GO derivatives less conductive, drastically compromising their potential as electrode materials. In principle, urgent and intense research efforts are required to develop optimized and reproducible synthesis approaches, aiming for effective graphene functionalization for improved electrode and device fabrication.

**Scheme 1:**
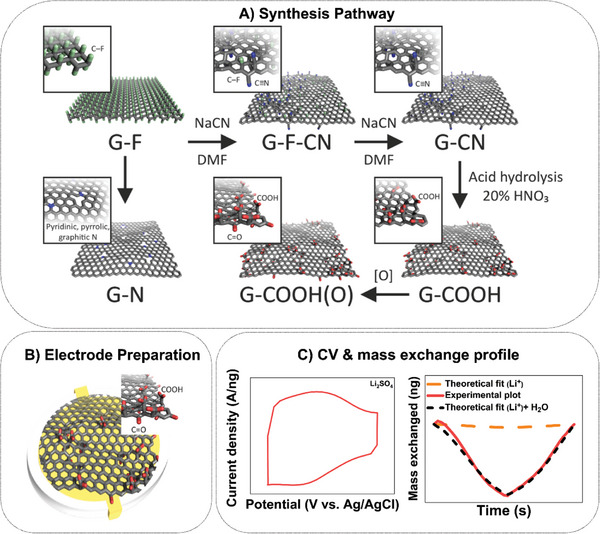
A) Synthesis route adopted for preparing multiple functionalized graphene derivatives (G–F = fluorographene, G–F–CN = fluorine‐doped cyanographene, G–CN = cyanographene, G–COOH = graphene acid, G–COOH (O) = oxidized graphene acid, G–N = nitrogen superdoped graphene) along with steps involved in the study of water‐assisted ion transfer behavior in the graphene derivatives in the order of B) electrode preparation, and C) cyclic voltammetry measurements and corresponding mass exchange profile procured from EQCM measurements.

The growing attention toward covalent synthesis strategies for fabricating multiple 2D graphene derivatives from a single parent has revolutionized the material fabrication approaches. Fluorographene (G–F),^[^ ^13^, ^41^, ^42^, ^43^, ^44^ ^]^ procured by fluorination of graphene followed by exfoliation is one systematic approach that is known to deliver stable graphene derivatives. It also has the potential to serve as the starting material to expand the graphene family by providing selective and densely functionalized derivatives of graphene.^[^ ^13^, ^15^ ^]^ Some interesting articles on fluorinated functionalized graphene are also available in the literature.^[^ ^45^, ^46^, ^47^, ^48^, ^49^ ^]^ “Cyanographene” (G–CN) is one such active material obtained by the reaction between sodium cyanide and G–F.^[^ ^50^ ^]^ During the reaction, the nucleophilic substitution of fluorine (─F) atoms by nitrile (─C≡N) groups occurs, and the extent of substitution is subject to the reaction conditions applied, for example, the reaction time.^[^ ^50^ ^]^ These variations in reaction conditions can lead to multiple intermediate products with varying degrees of ─F and ─C≡N functionalities, herein the derivative is termed as “fluorine‐doped cyanographene” (G–F–CN). Parallelly, the grafting of carboxylic groups (─COOH) directly on the graphene surface can be tedious, such as in GO, where it results in the functionalization of multiple oxygen (─O) containing groups and lower selectivity. Interestingly, selective and dense functionalization of the ─COOH groups over the graphene surface was an outcome of mild acidic hydrolysis of G–CN samples. Successful synthesis results in the formation of 2D acid, called “graphene acid” (G–COOH), with a homogeneously functionalized ─COOH graphene derivative.^[^ ^13^, ^50^, ^51^, ^52^ ^]^ Employing such covalent grafting of ─COOH groups over the graphene surface is known to enhance the interfacial properties of the material and is substantially more conductive than GO, while allowing electronic communication between the carboxyl groups and the aromatic backbone.^[^ ^51^ ^]^ Further, the oxidation of G–COOH results in another set of graphene derivatives called “oxidized graphene acid” and is termed as G–COOH (O). The material is functionalized with extra oxygen‐containing groups (carbonyl mainly) along with the ─COOH groups as reported previously.^[^ ^53^ ^]^ Accounting to a systematic study of various covalent functionalities over graphene, a “nitrogen (N) superdoped” graphene derivative (termed as G–N) was also synthesized along with other functional derivatives to understand the influence of ─N groups for ion storage. These were procured via conducting a reaction between G–F and sodium azide in the presence of dimethylformamide, where sodium azide acts as a defluorinating agent, and also as a ─N source for functionalization over the material surface.^[^ ^54^ ^]^ A detailed synthesis strategy is given in the Experimental Section. Interestingly, evaluating the electrochemistry of these graphene derivatives (**Scheme** **1**A) procured by a step‐by‐step chemical modification of the parent material (G–F) would be of interest to understand the potential of covalently functionalized graphene derivatives (G─X, where X = ─C≡N (cyanographene), ─F─C≡N (fluorine‐doped cyanographene), ─COOH (graphene acid), ─COOH (O) (oxidized graphene acid), ─N (nitrogen superdoped graphene)) for ion storage in various electrolyte solution.

Insight on the structural and dynamic properties of electrolyte solutions during the electrochemical process is also vital for device fabrication. In a study by Ohba et al.,^[^ ^55^ ^]^ water‐assisted ion transfer in graphene layers was studied which provided some interesting insights into the variation in hydration numbers, hydrogen bonding, and ion transfer during the charge‐discharge process. Also, the potential of the confined water molecules in carbon‐based nanostructure is well known^[^ ^56^, ^57^, ^58^ ^]^ and such an understanding on aqueous carbon nanofluidic phenomena can be vital for broadening the scope of covalently functionalized graphene‐based materials for a myriad of applications. Concurrently in the present context, the influence of varying surface functionalities on the critical role of water molecules assisting respective metal ions during the electrochemical process is under the purview of serious research. In short, the ion storage ability of layered material can be influenced by functional groups and assisted water molecules, wherein the water molecules can stabilize the layered structure by forming bridges between the graphene layer eventually enhancing the ion storage property.

Employing advanced characterization techniques, like in situ gravimetric electrochemical quartz–crystal microbalance (EQCM),^[^ ^59^, ^60^, ^61^, ^62^ ^]^ can aid toward procuring a real‐time probe of the compositional changes and understanding the charging mechanisms in graphene derivative at nanogram‐level sensitivity.^[^ ^63^ ^]^ High sensitivity and non‐destructiveness are added advantages of EQCM. Apart from the real‐time mass exchange of mobile active species at the electrode/electrolyte interface, information on the exchange of ions and possible solvent molecules can be extracted systematically and evaluated. Further, studies on aqueous‐based electrolytes are interesting owing to their low‐cost, non‐flammability, high power density, and a much safer handling system.^[^ ^64^, ^65^ ^]^ Also, studies on aqueous electrolyte systems have been reported on the transfer of water molecules along with active ionic species in the electrolyte using EQCM.^[^ ^62^, ^66^ ^]^

In the current study, the effect of functional terminations on the ion storage capability of Li^+^, Na^+^, and K^+^ ions and role of assisted water molecules in various graphene derivatives of G–F–CN, G–CN, G–COOH, G–COOH (O) and G–N is investigated. Real‐time tracking of the charge and simultaneous mass exchanged during the electrochemical process provided a deeper insight into these graphene derivatives. Computational chemistry was utilized to provide further insights into the observed phenomena and differences. It was observed that G–COOH (O) and G–COOH exhibited enhanced charge storage properties when compared to other sets of graphene derivatives such as G–F–CN, G–CN, and G–N. The transfer of water molecules in different cationic and anionic aqueous electrolyte systems (Li_2_SO_4_, Na_2_SO_4_, K_2_SO_4,_ LiCl, NaCl, and KCl) is elucidated, which brings a deeper understanding about the electrochemistry of various graphene derivatives and, thus, broadens their scope for energy storage applications.

## 2 Results and Discussion

### 2.1 Characterization of Graphene Derivatives

The synthesis protocol of all graphene derivatives is detailed in Scheme 1A. The nucleophilic reaction between cyanide (CN^−^) anions and fluorographene (G–F) results in the replacement of fluorine (─F) atoms by nitrile (C≡N) groups.^[^ ^50^ ^]^ The content in C–F groups and the F replacement by the C≡N groups can be controlled by the reaction time between CN^−^ anions and G–F, resulting in fluorine‐doped cyanographene (G–F–CN) or to almost fully defluorinated cyanographene,^[^ ^43^, ^50^ ^]^ which will be discussed in detail later. The morphology of the samples was evaluated by scanning electron microscopy (SEM). G–F–CN shows a layered lump‐like morphology with granular protrusions throughout the sample surface as shown in Figure S1A (Supporting Information). Cyanographene (G‐CN) showed a similar lump‐like stacked structure, identical to G–F‐CN, however, no granular protrusions were identified on the material surface (Figure S1B, Supporting Information). Energy dispersive X‐ray spectroscopy (EDX) was employed to evaluate the elemental distribution in G–F–CN (Figure S2A, Supporting Information) and G–CN samples (Figure S2B, Supporting Information). Both samples showed the uniform distribution of carbon (C), nitrogen (N), and oxygen (O) while ─F was more evident in G–F–CN electrode material (Figure S2A,B, Supporting Information). X‐ray photoelectron spectroscopy (XPS) was performed to investigate the elemental composition of the synthesized samples. The survey spectrum of synthesized graphene derivatives is given in Figure S3A (Supporting Information). The G–F–CN samples showed peaks of C, O, and N at binding energy (BE) of 284.6 eV, O at 532.6 eV, N at 399.6 eV. Also, ‐F peak at BE of 687.6 eV affirms the presence of F dopants in the G–F‐CN sample. Quantitative analysis of the sample showed that the atomic percentage (at.%) of C 1s, O 1s, N 1s, and F 1s is in the order of 80.79%, 4.2%, 8.5%, and 6.4%, respectively. Meanwhile, the XPS survey of pristine G–F is depicted in Figure S3A (Supporting Information), wherein the at.% of C 1s, O 1s, and F 1s are in the order of 48.4%, 1.1%, and 50.5%, respectively. The ─F content in the derivatives substantially reduced from the initial 50.5 at.%, evident of the simultaneous defluorination and replacement of ─F with ─C≡N groups. Further, the quantitative XPS analysis of G–CN samples gave peaks of C 1s, O 1s, N 1s with at.% procured in the order of 80.91%, 5.05%, and 13.4%, respectively. A steep decline in the ─F peak is observed in G–CN derivatives when compared to G–F‐CN sample, wherein at.% of ─F decreased from 6.4% (G–F‐CN) to 0.64% (G‐CN) clearly confirms the substantial removal of ─F functionalities from the graphene derivatives subjected to specific reaction conditions. Deconvoluted C 1s spectra of G–CN (Figure S3B, Supporting Information) samples confirmed a significant component of aromatic sp^2^ carbon atoms. Fourier transform infrared (FTIR) spectra of G–F, G–F‐CN, and G‐CN samples were also measured as shown in Figure S3C (Supporting Information).

It was observed that the relative area of the C≡N band was higher in G‐CN samples, compared to G–F‐CN samples at around 2200 cm^−1^. Further, a decrease in ─F residues in G–CN results in an increased sp^2^ network, which is reflected by higher relative intensity and area of C═C band at 1500–1600 cm^−1^ (Figure S3C, Supporting Information) compared to G–F‐CN samples. The sp^2^ network band is completely absent in pristine G–F, and only the CF (at 1200 cm^−1^) and CF_2_ (at 1320 cm^−1^) bands are evident. Thus, the above characterization clearly affirms the decline in ─F residues in G–CN samples and is decorated with C≡N groups throughout the graphene network. Further, Raman analysis of electrode materials was employed to evaluate the carbon structure and functionalization of electrode material. The G–CN (Figure S4, Supporting Information) derivative showcased an intense defective peak confirming that the functionalization is successful^[^ ^50^ ^]^ (detailed in the supporting information).

Further, graphene acid (G–COOH) was procured from hydrolysis of G–CN based on the previously reported methods.^[^ ^50^ ^]^ Upon mild acidic hydrolysis, C≡N covalent functionalities are converted to ─COOH, yielding G–COOH.^[^ ^50^, ^51^ ^]^ The covalently grafted ─COOH groups over the conductive graphene materials aid in the fabrication of efficient and stable electrode material for energy storage applications.^[^ ^51^ ^]^ The material is known to exhibit high dispersibility in water media, high conductivity, and biocompatibility.^[^ ^43^, ^50^ ^]^ This approach is highly advantageous pertaining to the fact that direct attachment of the ─COOH group over graphene via other methods is tedious and involves harsh oxidation conditions. Hence, it may not lead to the functionalization with the desired groups but to diverse ─O containing terminations.^[^ ^67^, ^68^ ^]^ Also, it was reported that ─O functionalities modify the interaction in graphene layers and contribute toward pseudocapacitances.^[^ ^69^, ^70^ ^]^ Hence, another graphene derivative, termed “oxidized graphene acid” (G‐COOH (O)) was also synthesized, which is procured by further oxidation of the G–COOH derivative.^[^ ^53^ ^]^ This approach introduces additional ─O functionalities (─C═O) along with the inherent COOH groups on the carbon lattice. Hence, employing a comparison between two materials, G–COOH and G–COOH (O), would help us draw some interesting observations on the influence of functionalities toward ion storage applications. The detailed synthesis protocol of G–COOH and G–COOH (O) is given in the Experimental Section. G–COOH (Figure S1C, Supporting Information) and G–COOH (O) (Figure S1D, Supporting Information) showcase a similar layered morphology according to SEM. EDX mapping of G–COOH (Figure S2C, Supporting Information) and G–COOH (O) (Figure S2D, Supporting Information) confirmed the presence of C, O, and N elements. Further, XPS on G–COOH and G–COOH (O) showed peaks of C, O, and N at respective BE. In G–COOH, the relative at.% of C, O, and N is in the order of 81.8%, 14.6%, and 2.9%, and in G–COOH (O) the at.% is in the order of 80.9%, 18.2%, 0.8%, respectively (Figure S3A, Supporting Information). The G‐COOH (O) displayed a slightly higher content in ─O due to the additional oxidation of G–COOH material. The absence of ─F terminations in G–COOH and G–COOH (O) affirms that the acid hydrolysis was successful, wherein –F termination is completely eliminated and ─CN groups are converted to ─COOH groups. Comparing deconvoluted C 1s spectra of G‐COOH (Figure S5A, Supporting Information) and G–COOH (O) (Figure S5C, Supporting Information) graphene derivative with C 1s spectra of G–CN (Figure S3B, Supporting Information) samples, the presence of carboxyl‐type (─COOH) carbons is evident in the two former samples, while no such peak was evident in the G‐CN sample. This clearly confirms that the acid hydrolysis approach was successful in fabricating material with varying carboxyl contents (G–COOH and G–COOH (O)). Also, comparing O 1s spectra of G–COOH (Figure S5B, Supporting Information) and G–COOH (O) (Figure S5D, Supporting Information) samples displayed two main components of ─C═O and ─C─OH peaks along with a third minor peak ascribed to chemisorbed O_2_ and H_2_O. Additionally, a relative increase in the ─C═O bond over the ─C─OH component indicates the possibility that higher HNO_3_ concentrations induced less selective oxidation of the graphene backbone. The XPS results are in line with the previous results available in the literature. The small amount of ‐N originates from the DMF solvent during the synthesis, as confirmed by control experiments with solvent only.^[^ ^50^ ^]^ Also, the Raman analysis confirmed the functionalization was successful in both G–COOH (Figure S4, Supporting Information) and G–COOH (O) (Figure S4, Supporting Information) materials. G–COOH and G–COOH(O) samples showcased a broadening of the Raman bands when compared to G–CN samples (detailed in the Supporting Information).

Furthermore, Figure S1E (Supporting Information) depicts the SEM image of G‐N graphene derivatives with stacked sheet‐like morphology procured by the covalent functionalization of N source over graphene material. Upon elemental mapping of the samples, as shown in Figure S2E (Supporting Information), the samples confirmed the N doping was successful along with the presence of C, O, and F elements. Further, the N doping in G‐N samples was confirmed from XPS analysis (Figure S3A, Supporting Information), wherein at.% of N was around 15.1% (highest N content among all the synthesized graphene derivatives).^[^ ^54^ ^]^ Also at.% of C 1s, O 1s, and F 1s were procured in the order of 80.0%, 3.0%, and 1.7%, respectively. Deconvoluted C 1s spectra of G‐N samples (Figure S5E, Supporting Information) showed peaks of aromatic sp^2^ carbon peaks, non‐functionalized sp^3^ carbons, nitrogen‐bonded carbons, and small residual atoms of fluorine and oxygen. The HR‐XPS spectra of the N 1s (Figure S5F, Supporting Information) displayed the presence of N atoms in pyridinic, pyrrolic, and graphitic configurations. Raman measurements confirmed the doping was successful with the intense D band over the G band as shown in Figure S4 (Supporting Information). Water contact angle measurements of graphene derivatives were carried out as well. Initially, the experiment was carried out on a bare silicon wafer substrate, and the water contact angle measured a value of 55.4^0^ (Figure S6A, Supporting Information). Further to analyze the water contact angle measurement of all samples, the graphene derivatives were well coated on a silicon wafer substrate. The contact angle measurements results of all assessed graphene‐based samples were below 20^0^ (Figure S6B–F, Supporting Information), showing the hydrophilic nature of the synthesized graphene derivatives. Accounting for this nature of electrode materials, the water spread quickly across the graphene‐coated material substrate forming a very thin film. In short, the above experiment has given a direct indication toward the hydrophilic nature of the graphene derivatives.

The specific surface area of all graphene derivatives was calculated using the Brunauer‐Emmett‐Teller (BET) method. Respective BET isotherm and pore distribution plots of all electrode materials are presented in Figure S7A‐L (Supporting Information). The surface area decreased with increasing functionalization. The BET surface area of the parent material (G–F) was observed to be 286 m^2^ g^−1^, while it decreased to 148, 20, 17.6, 9.3, and 87.2 m^2^ g^−1^ for G–F–CN, G–CN, G–COOH, G–COOH (O), and G–N electrode materials, respectively. Furthermore, analyzing the porosity of active graphene derivatives showed that all electrode materials had a mesoporous structure.

### 2.2 Electrochemistry of Graphene Derivatives

Following characterization of the graphene derivatives, electrochemical measurements were carried out systematically to evaluate the charge storage performance of these active materials. Primarily, cyclic voltammetry (CV) measurements were carried out in Li_2_SO_4_, Na_2_SO_4_, and K_2_SO_4_ electrolyte solutions of similar concentrations and anions (SO_4_^2−^) but with differing cations (Li^+^, Na^+^, K^+^). By employing real‐time monitoring of gravimetric probes EQCM helps evaluate the ion storage behavior of the active material along with mass exchange.^[^ ^26^, ^66^ ^]^ In short, the samples were spray‐coated to procure a uniform film of identical thickness on gold (Au) quartz crystal (Scheme 1B), which served as an ideal substrate. Based on the electrochemical reaction between the electrolyte and electrode material, resonant frequency changes (Δ*f*) are procured with respect to the CV measurements (Scheme 1C). Employing EQCM as a gravimetric probe, Δ*f* can be simultaneously translated into the electrode mass changes (Δ*m*) using the Sauerbrey equation, which is given by Δ*m* = −C*Δ*f*/*n*, where C is the calibration constant (or sensitivity factor), which is 17.7 ng cm^−2^ Hz^−1^ and *n* = 1.^[^ ^62^, ^71^ ^]^ Evaluating the mass exchange profile of the electrode material associated with each electrolyte system helps to procure information on the exchanged species during the electrochemical process. For instance, real‐time tracking of solvated/non‐solvated, ionic, and/or non‐ionic species exchanged can be identified using this technique, which eventually would provide us with a better understanding of the charge storage performance of the active electrode graphene derivatives. Studies on EQCM for supercapacitor applications is often valuable. For instance, QCM was employed to monitor ion flux in microporous activated carbon materials,^[^ ^72^ ^]^ thereby enhancing their scope in devising newer electrode materials for supercapacitor applications. In the context of 2D materials interesting studies have also been reported, where EQCM was used to study the real‐time ion transport behavior of graphene channels with varying 2D channel spacing^[^ ^73^ ^]^ and the possibilities of anion insertion into the restacked MXene electrodes.^[^ ^25^ ^]^ Also, very recently the role of N doping in the charging mechanism in porous carbon materials was reported using EQCM and solid‐state NMR studies.^[^ ^74^ ^]^ These studies clearly illustrate the importance of EQCM in the study of the charging mechanism of supercapacitors. Thus, for the first time, in the current study, the influence of functional groups in covalently functionalized graphene derivatives is studied and analyzed systematically.

**Figure 1:**
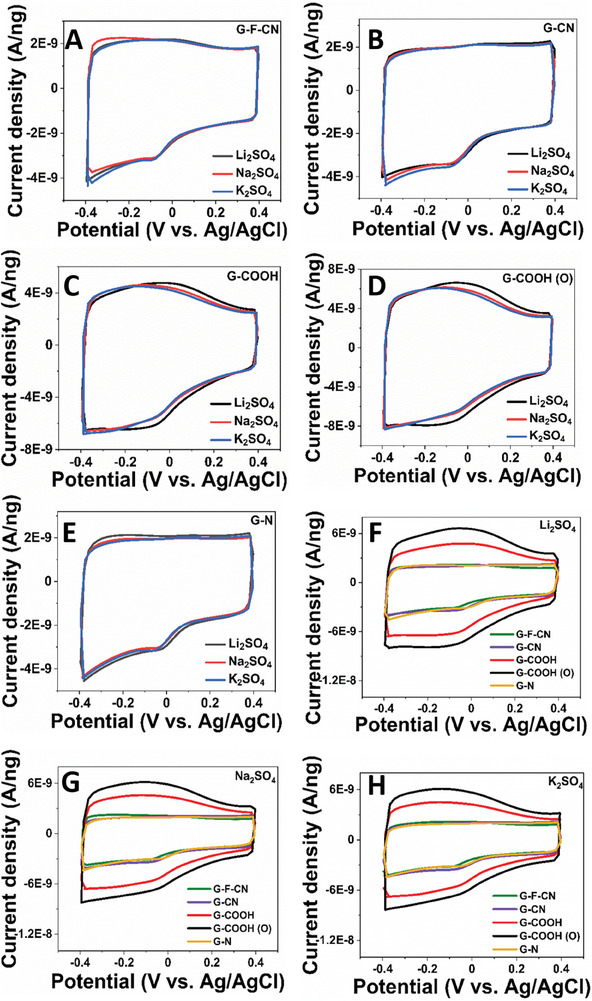
CV profile of A) fluorine‐doped cyanographene, B) cyanographene C) graphene acid, D) oxidized graphene acid, and E) nitrogen superdoped graphene samples in various electrolyte systems. CV measurements of graphene derivatives in 0.5 m F) Li_2_SO_4_ G) Na_2_SO_4_ and H) K_2_SO_4_ electrolyte solutions at a scan rate of 50 mV s^−1^.

**Figure** **1**A–E shows the comparative CV profile measurements of G–F–CN, G–CN, G–COOH, G–COOH (O), and G–N electrode materials, respectively, in 0.5 m Li_2_SO_4_, Na_2_SO_4_, and K_2_SO_4_ electrolyte systems. It was observed that all graphene derivatives namely G–F–CN (Figure 1A), G–CN (Figure 1B), G–COOH (Figure 1C), and G–COOH (O) (Figure 1D), and G–N samples (Figure 1E) showed a very similar current response in three electrolyte solutions of Li_2_SO_4_, Na_2_SO_4_, and K_2_SO_4_. A rectangular‐shaped CV profile was procured in all three electrolyte systems, that can be correlated to the reversible electroadsorption/desorption of ions in the electrolyte, and minor deviations in the CV profile can be correlated to the faradaic contribution from functional groups during the CV measurement.

Further, the influence of covalent functionalization of five synthesized graphene derivatives toward charge storage was evaluated in electrolyte solutions of 0.5 m Li_2_SO_4_ (Figure 1F), Na_2_SO_4_ (Figure 1G), and K_2_SO_4_ (Figure 1H). It was observed that G–COOH (O) and G–COOH exhibited a close ion storage response in all three electrolyte systems, with the former being slightly higher. Meanwhile, low capacitance current was procured in the other graphene derivatives, in the order of G–COOH (O) > G‐COOH > G–N ≈G–F–CN ≈G–CN. In G–COOH, the aromatic conjugated carboxylic derivative exhibits an intramolecular charge delocalization property, making it ideal for charge storage, outweighing other graphene derivatives. Some interesting reports are also available in the literature.^[^ ^75^, ^76^, ^77^ ^]^ Furthermore, excessive oxidation of G–COOH results in the formation of G–COOH (O), with ─O terminations on the carbon surface along with the ─COOH functionalities, which enhance the charge storage performance of the active material. Enhanced hydrophilicity rendered by ─O terminations of ─C═O and ─COOH functionalities can also cause the solvent water molecules in the electrolyte to form hydrogen‐bonded bridges between the carbon layers, eventually broadening the possibilities for higher ion storage. Reductive defluorination of G–F–CN samples along with replacing ─F with ─C≡N aided toward G–CN samples, which is the starting material for G–COOH synthesis.^[^ ^50^ ^]^ It was observed that both G–F‐CN and G‐CN delivered very low capacitance current compared to other graphene derivatives studied here. Replacing ─C≡N with ─COOH to procure G–COOH is known to enhance the ionic conductivity of the active material, allowing facile movement of the charge carriers and ions along the electrode material and making them ideal for charge storage applications.^[^ ^51^ ^]^

### 2.3 Water Assisted Cation Transfer

**Figure 2:**
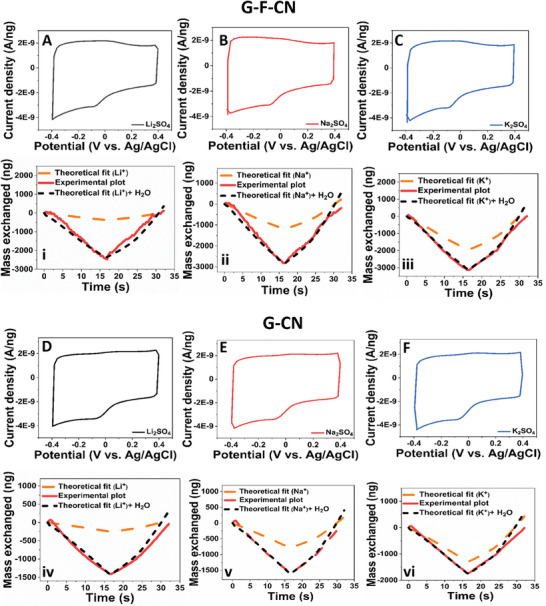
A–C) CV and mass exchange profiles of fluorine‐doped cyanographene (G–F–CN) sample in 0.5 M Li_2_SO_4_ A,i), Na_2_SO_4_ B,ii), and K_2_SO_4_ C,iii), respectively. D–F) CV and mass exchange profiles of cyanographene (G–CN) sample in 0.5 m Li_2_SO_4_ D,iv), Na_2_SO_4_ E,v), and K_2_SO_4_ F,vi), respectively.

EQCM is employed for the real‐time tracking of exchanged active ionic species during the CV measurements in various electrolyte solutions. **Figure** **2** shows the CV profile of G–F‐CN electrode material in 0.5 m Li_2_SO_4_ (Figure 2A), 0.5 m Na_2_SO_4_ (Figure 2B), and 0.5 m K_2_SO_4_ (Figure 2C), respectively at scan rate of 50 mV s^−1^. Corresponding to each CV profile, the mass exchange plot is evaluated based on the real‐time tracking of species interfacial transfer during an electrochemical process. It revealed a cationic exchange during the electrochemical process in all the studied graphene derivatives. Thus, in the present context, the theoretical mass exchange is calculated by considering only the ionic species (bare cation) exchanged during an electrochemical process. Specifically, Li^+^ (7 g mol^−1^), Na^+^ (23 g mol^−1^), K^+^ (39 g mol^−1^) and the number of moles are procured from the CV profile. Further, in the case of G–F–CN electrode material in 0.5 m Li_2_SO_4_ electrolyte solution, it was observed that the total mass exchange (experimental plot) was higher than the theoretical mass calculated from bare Li^+^ exchanged during an electrochemical process (Figure 2‐i). In other words, if only the transfer of non‐hydrated species was involved during the CV, then the corresponding mass exchange would correspond exclusively to the number of ions. However, this excessive mass can be correlated to the water molecules in the electrolyte that aid toward the hydration of Li^+^ cations and/or are exchanged along with the cations in the free form. Thus, such information on the species exchanged during the electrochemical process can be procured with the following technique. To calculate the intrinsic water exchanged during the electrochemical process, the following equation can be employed

(1) $$\begin{array}{ccc} \mathrm{Water}\;\mathrm{exchanged}\left(x\right) & = & \left(M_{\left(\mathrm{theoretical}\;\mathrm{fit}\left({\mathrm{Li}}^{+}/{\mathrm{Na}}^{+}/K^{+}\right)+x.H_{2}O\right)}\right.\\ & & -\left.M_{\left(\mathrm{theoretical}\;\mathrm{fit}\left({\mathrm{Li}}^{+}/{\mathrm{Na}}^{+}/K^{+}\right)\right)}\;\right)\;/M_{\left(H_{2}O\right)} \end{array}$$

where, $M_{\left(\mathrm{theoretical}\;\mathrm{fit}\left({\mathrm{Li}}^{+}/{\mathrm{Na}}^{+}/K^{+}\right)+x.H_{2}O\right)}$ = sum total of molar mass of exchanged species (ions/water molecules), M _(theoretical fit (Li+/Na+/K+)_ = molar mass of respective cations in the systems (Li^+^/Na^+^/K^+^), M _(H2O)_ corresponds to molar mass of water (18 g mol^−1^). The above calculation strategies are employed for all the studied graphene derivatives.

Further, upon analyzing the mass exchange profiles of G–F‐CN electrode samples in Na_2_SO_4_ (**Figure 2**‐ii) and K_2_SO_4_ (Figure 2‐iii) electrolyte systems, it was observed that total mass exchange was higher than the theoretical mass calculated from bare Na^+^ (Figure 2‐ii) and bare K^+^ (Figure 2‐iii) during the electrochemical process. The CV profile of G–F‐CN electrode material in 0.5 m Li_2_SO_4_ at 20 and 100 mV s^−1^ and its associated mass exchange profile are shown in Figure S8A,B,C (Supporting Information), respectively. Further, evaluating the molecular mass calculations procured from EQCM measurements of exchanged species, the total mass procured was in the order of 42–58 g mol^−1^ for aqueous Li_2_SO_4_ systems, wherein, these molar masses belong to the molar mass of respective ionic species and exchanged water molecules. In this context of G–F‐CN derivative, corresponding content ratio of Li^+^/H_2_O was obtained in the order of 2.77, 2.11, and 2 at scan rate of 20, 50, and 100 mV s^−1^, respectively.

Similarly, the CV profile of G–F–CN electrode material and mass exchange profile in Na_2_SO_4_ (Figure S8D–F, Supporting Information) and K_2_SO_4_ (Figure S8G–I, Supporting Information) at 20 and 100 mV s^−1^ scan rates are evaluated. The total mass procured was in the order of 45–66 g mol^−1^ for aqueous Na_2_SO_4_ systems and 56–82 g mol^−1^ for aqueous K_2_SO_4_ systems. Further, the associated exchanged water molecules corresponding to the content ratio of Na^+^/H_2_O and K^+^/H_2_O at 20, 50, and 100 mV s^−1^ is given in Table S1 (Supporting Information), T (a).

With respect to the graphene derivative G–CN, CV measurements were evaluated to understand the effect of C≡N functional groups as G–CN serves as a bridge between G–F–CN and G–COOH electrode materials. Figure 2D–F shows CV profile and mass exchanged plots for G–CN samples in various electrolyte solutions of 0.5 m Li_2_SO_4_ (Figure 2 D, iv), Na_2_SO_4_ (Figure 2 E, v), and K_2_SO_4_ (Figure 2F, vi) at scan rate of 50 mV s^−1^. Further, the associated CV profile and mass exchanged plots for G–CN samples at 20 and 100 mV s^−1^ scan rate in 0.5 M Li_2_SO_4_ electrolyte solution are given Figures S9A–C (Supporting Information), Na_2_SO_4_ (Figure S9D–F, Supporting Information), and K_2_SO_4_ (Figure S9G–I, Supporting Information), respectively. The mass of exchanged species is found to be in the order of 37–50 g mol^−1^ for aqueous Li_2_SO_4_ systems, 45–57 g mol^−1^ for aqueous Na_2_SO_4_ systems, and 44–64 g mol^−1^ for aqueous K_2_SO_4_ systems. The information of the ratio of Li^+^/H_2_O, Na^+^/H_2_O, and K^+^/H_2_O is given in supporting information Table S1 (Supporting Information), T (b), procured from the mass exchange profile of each system.

**Figure 3:**
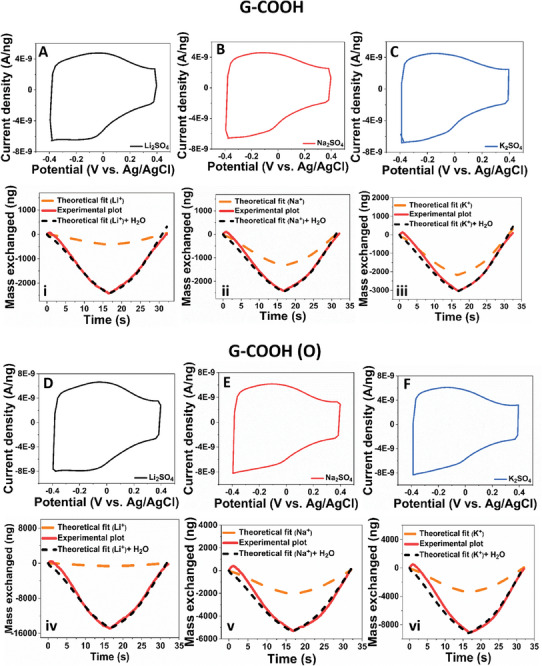
A–C) CV and mass exchange profiles of graphene acid (G–COOH) sample in 0.5 m Li_2_SO_4_ A, i), Na_2_SO_4_ B, ii), and K_2_SO_4_ C, iii), respectively. Figure D–F) CV and mass exchange profiles of oxidized graphene acid (G–COOH (O))  sample in 0.5 m Li_2_SO_4_ D, iv), Na_2_SO_4_ E, v), and K_2_SO_4_ F, vi), respectively.

In the next step of assessment, the influence of –O terminations in –COOH is evaluated for ion storage behavior. **Figure** **3** shows the CV and associated mass exchange profiles of G–COOH electrode material at 50 mV s^−1^ in various electrolyte solutions of 0.5 m Li_2_SO_4_ (Figure 3A, i), Na_2_SO_4_ (Figure 3B, ii), and K_2_SO_4_ (Figure 3C, iii), respectively. Upon, tracking the mass exchanged between the electrode/electrolyte interface in G–COOH during electrochemical CV measurement the influence of exchanged water molecules is clearly evident, like in the G–F–CN and G–CN electrode systems. A similar trend was observed upon evaluating the charge storage ability of material at scan rates of 20 and 100 mV s^−1^, respectively. The associated CV profile and mass exchanged plots for G–COOH in 0.5 m Li_2_SO_4_ electrolyte solution at 20 and 100 mV s^−1^ are given in Figure S10A–C (Supporting Information), Na_2_SO_4_ (Figure S10D–F, Supporting Information), and K_2_SO_4_ (Figure S10G–I, Supporting Information), respectively. The mass of exchanged species is found to be in the order of 35–45 g mol^−1^ for aqueous Li_2_SO_4_ systems, 35–48 g mol^−1^ for aqueous Na_2_SO_4_ systems, and 53–70 g mol^−1^ for aqueous K_2_SO_4_ systems. Further, calculating the exchange of water molecules along with each cation species from the mass exchange profile of each system, the procured details of Li^+^/H_2_O, Na^+^/H_2_O, and K^+^/H_2_O are given in Table S1 (Supporting Information), T (c).

Further, in the oxidized 2D acid samples, Figure 3D–F shows the CV and associated mass exchange profiles of G–COOH (O) electrode material in solutions of 0.5 m Li_2_SO_4_ (Figure 3D‐ iv), Na_2_SO_4_ (Figure 3E‐v), and K_2_SO_4_ (Figure 3F‐vi), respectively at 50 mV s^−1^. The CV profile and mass exchanged plots of G–COOH (O) in 0.5 m Li_2_SO_4_ (Figure S11A–C, Supporting Information), 0.5 m Na_2_SO_4_ (Figure S11D–F, Supporting Information), and 0.5 m K_2_SO_4_ (Figure S11G–I, Supporting Information) are measured at 20 and 100 mV s^−1^ scan rates. However, upon careful evaluation of the mass exchanged in all three electrolyte systems, it was observed that the final masses obtained were higher than the theoretical molar mass of cations in the electrolyte, similar to the one observed in G–F–CN, G–CN and G–COOH samples. Hence, the exchange of both cation and associated water molecules was clearly identified using the EQCM technique. The total mass exchange obtained is in the order of 155–168, 56–71 and 84–121 g mol^−1^ for Li^+^, Na^+^, and K^+^ systems, respectively. The associated cation/water molecule transferred in each system (procured from the mass exchange profile of each system) at scan rate of 20, 50, and 100 mV s^−1^ are given in supporting information Table S1 (Supporting Information), T (d). It is observed that very high mass exchange was evident in the Li^+^ systems of G–COOH (O) electrode material. This very high enhancement can be corroborated to the excessive hydrophilic nature of oxidized graphene derivative, attracting a very high amount of water molecules, which could both be in free form or assisted with Li^+^ cation.

**Figure 4:**
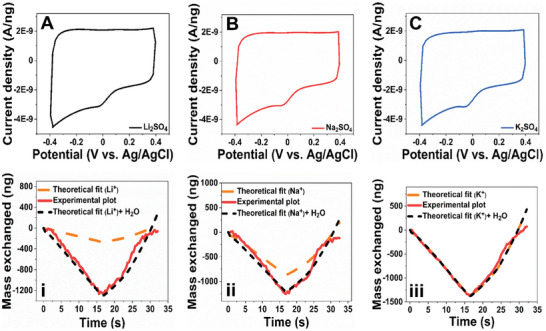
CV and mass exchange profiles of nitrogen superdoped (G–N) graphene samples in 0.5 m Li_2_SO_4_ A, i), Na_2_SO_4_ B, ii), and K_2_SO_4_ C, iii), respectively.

With the objective to evaluate the influence of ─N doped functionalized graphene derivative toward ion storage, and respective real‐time mass exchange profiles of G–N electrode material was procured in solutions of 0.5 m Li_2_SO_4_ (**Figure** **4**A, i), Na_2_SO_4_ (Figure 4B‐ii), and K_2_SO_4_ (Figure 4C‐iii), respectively at 50 mV s^−1^. The exchange of water molecules was evident, affirming the fact that all functionalized graphene derivatives showcased the water‐assisted ion transfer behavior. The CV profile and mass exchanged plots at 20 and 100 mV s^−1^ scan rates in 0.5 m Li_2_SO_4_ (Figure S12A–C, Supporting Information), 0.5 m Na_2_SO_4_ (Figure S12D–F, Supporting Information), and 0.5 m K_2_SO_4_ (Figures S12G–I, Supporting Information) are measured and evaluated as well. The total mass exchange obtained is in the order of 31–48, 28–46 and 39–48 g mol^−1^ for Li^+^, Na^+^, and K^+^ systems, respectively. Further, Table S1 (Supporting Information), T (e), details the cation/water molecule transferred in each system at scan rates of 20, 50, and 100 mV s^−1^.

**Figure 5:**
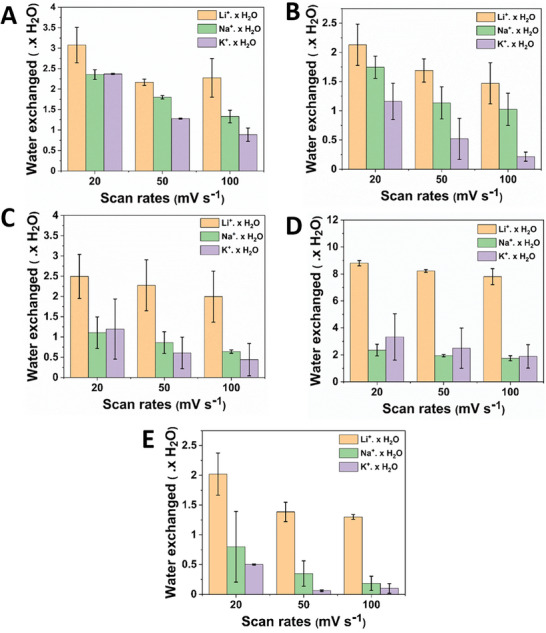
Trend procured in exchange of water molecules with respect to scan rates in A) fluorine‐doped cyanographene; B) cyanographene; C) graphene acid; (D) oxidized graphene acid; E) nitrogen superdoped graphene, respectively.

A closer inspection of the water molecules in the studied graphene derivatives on repetition with respect to scan rate revealed an interesting pattern. The number of water molecules accompanying the cations during the CV measurements was inversely related to the scan rates (**Figure** **5**A–E). For instance, in all the studied graphene derivatives, the exchange of water molecules decreased in the order of 20, 50, and 100 mV s^−1^, respectively. Upon comparing G–F–CN and G–CN graphene derivatives, the water exchange is found to be in close proximity with respect to corresponding Li^+^, Na^+^, and K^+^ systems, although the former exhibits slightly higher exchange. CV profile of these derivatives (Figure 1F–H) showed a very close ion storage behavior for Li^+^, Na^+^, and K^+^ systems. Thus, ─F termination combined with ─C≡N functionalities and/or derivatives with ─C≡N alone, displays a similar charge storage property, affirming no major visible differences arising from functionalities, except for higher water exchange in systems with dual functionalities, such as in G–F‐CN (─C≡N and ─F). From the CV profile (Figure 1F–H) of graphene derivatives, G‐N showcased similar charge storage performance like G–F–CN and G–CN derivatives, but comparatively much lower water exchange.

Interestingly, G–COOH (O) electrode material showed high water exchange when compared to G–COOH, G‐N, G–F–CN, G–CN electrode material (Figure 5A–E). This can be attributed to the excessive oxygenated species on the graphene derivatives, such as ─O terminations, ─COOH functionalities, ─C═O groups, resulting in higher water exchange. The high‐water exchange during the electrochemical process in G–COOH (O) electrode material is assumed to result in stabilizing the layered structure, and thus outperforming all the other graphene derivatives in ion storage (Figure 1F–H). In short, ─O containing graphene derivatives (G–COOH and G–COOH (O)), showcase high ion storage behavior with respect to other graphene derivatives with functional groups of ‐C≡N, ─N, ─F, and with mixed functionalities of ─F and ─C≡N. However, excessive oxidation of 2D acid derivatives results in mixed functionalities of ─O terminated ─COOH and carbonyl‐based active groups resulting in excess hydrophilic nature^[^ ^53^ ^]^ followed by high water‐assisted ion transfer. Further, reports on the water uptake in graphene‐based materials are available in literature.^[^ ^53^, ^78^ ^]^ The exchanged water molecules are expected to stabilize the layered material by occupying the nanoconfined spaces/traps within the material and resulting in highly conductive, and intact electrode material for ion storage applications.^[^ ^27^ ^]^ In other words, the assisted water molecules during ion transfer bridges with ─O functionalities between graphene layers, eventually delivering a high current response when compared to other sets of graphene derivatives. In principle, the evaluation of water molecules transferred alongside Li^+^ ions in the examined graphene derivatives revealed the order of hydration numbers and/or assisted water molecules was found to be 2.8, 2.4, 2.1, 8.9, and 2.3 for G–F–CN, G–CN, G–COOH, G–COOH (O), and G–N, respectively (Table S1, Supporting Information). Interestingly, they are close to those reported on 2D germanane‐MXene heterostructure of 3.5,^[^ ^62^ ^]^ 2D Ti_3_C_2_ MXene of 5.2,^[^ ^26^ ^]^ and 3 reported for electrochemically reduced graphene oxide thin film electrodes.^[^ ^66^ ^]^ Overall, the hydration number and/or the extent of water‐assisted ion transfer depend namely on the electrode material, surface functionalities, scan rate, electrolyte solutions etc.

Interestingly, upon comparing the surface area results with the electrochemistry data, it was noted that although the surface area decreased with increasing functionalization in the order of G–F→ G–F–CN→ G‐CN→ G–COOH→ G–COOH (O), it exhibited a high charge storage performance. This implies that although the available active sites for charge storage decrease with increased functionalization, the available sites are highly efficient for charge storage. Such functionalization strategies can lead to the fabrication of the lightweight and/or compact energy storage devices for future energy storage applications. Furthermore, the role of assisted water molecules is analyzed in the following study, opening up newer possibilities of electrode fabrication for energy storage devices as these electrode materials can absorb and hold significant amounts of electrolyte due to hydrophilic nature, increasing the capacitance and charge storage ability of graphene‐based electrode materials.

The analysis of surface hydrophilicity through contact angle measurements provides an indirect indicator of the surface's affinity for water. Conversely, EQCM technique affords a deeper understanding by examining the ease with which water and ions permeate the interstitial spaces of graphene layers. This distinction underscores the existence of two different interfaces that water molecules probe in the two techniques. The first interface explored by EQCM is dominated by the interactions within the functionalized graphene layers, highlighting the material's internal dynamics. In contrast, contact angle measurements elucidate the external interface between functionalized graphene and air, focusing on the efficiency with which water molecules displace air at the surface.

Electrochemical impedance spectroscopy (EIS) measurements of the samples were also carried out to better understand the electrochemical properties of the synthesized samples (Figure S13, Supporting Information). The experiments were conducted using the three‐electrode setup in 0.5 m Li_2_SO_4_ electrolyte solution and were recorded at the open‐circuit potential. The G–CN sample displayed much higher charge transfer properties compared to G–F‐CN (Figure S13A, Supporting Information) demonstrating the enhancement in the conductivity upon nucleophilic substitution of ─F atoms by C≡N and restoration of the aromatic network. G–COOH and G–COOH (O) also showcased improved charge transfer over the G–N electrode material. These results were in line with the produced current densities recorded in the respective CVs (Figure 1F–H). Evaluating the performance of G–CN and G–COOH electrode materials, although G–CN displayed improved charge transfer properties (in line with previous results on electron conductivity of 500 S m^−1^ for G–CN 25 S m^−1^ for G–COOH)^[^ ^50^ ^]^, the G–COOH electrode materials delivered higher current densities over G–CN. These results further highlight the importance of functionalization and type of functional groups for creating highly efficient sites for charge storage via improving the electrode's solvation in the electrolyte, as revealed from EQCM measurements. It should be also noted that the degree of functionalization in such fluorographene derivatives might also play an important role in the energy storage properties.^[^ ^79^ ^]^

### 2.4 Ion Storage Subjected to Change in Electrolyte Anion

**Figure 6:**
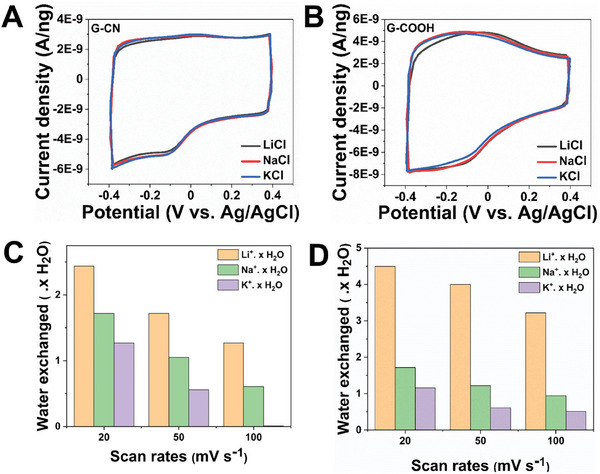
CV profile of A) G–CN and B) G–COOH in an electrolyte solution of LiCl, NaCl, and KCl respectively. Trend procured in exchange of water molecules with respect to scan rates in C) G–CN D) G–COOH electrode material.

Further, electrochemical performance of graphene derivatives was evaluated in electrolyte solution other than sulfate anions (SO_4_^2−^). EQCM studies of two graphene derivatives namely G–CN and G–COOH were performed in electrolyte solution of 0.5 m of LiCl, NaCl, and KCl. **Figure** **6**A,B shows the CV profile of G–CN and G–COOH electrode material in various electrolyte solutions respectively at scan rate of 50 mV s^−1^. Clearly, the G–COOH graphene derivative displays a higher charge capacitance over G–CN electrode material, which is in line with the results procured from SO_4_^2−^ electrolyte systems. Interestingly, a slightly higher capacitance current is evident in chloride (Cl^−^) systems when compared to SO_4_^2−^ electrolyte medium (Figure S14A–F, Supporting Information).

CV and mass exchange response of G–CN electrode material at scan rates of 20, 50, and 100 mV s^−1^ was recorded in an electrolyte solution of 0.5 m LiCl (Figure S15A–D, Supporting Information), 0.5 m NaCl (Figure S16A–D, Supporting Information), and 0.5 m KCl (Figure S17A–D, Supporting Information), respectively. It was noted that in all the three systems, cation‐assisted water molecule transfer was evident during the electrochemical process. The total mass exchange obtained is in the order of 30–51, 34–54 and 39–62 g mol^−1^ for Li^+^, Na^+^, and K^+^ systems, respectively. The trend in water exchange is comparable to the results procured in the sulfate systems. Similarly, the CV and corresponding mass exchange profile at respective scan rates were recorded in electrolyte solution using the G‐COOH graphene derivative. Figure S18A–D (Supporting Information) depicts the CV and mass exchange profile of G–COOH graphene derivative in 0.5 m LiCl, while Figure S19A–D, and S20A–D (Supporting Information), accounts to 0.5 m NaCl and 0.5 m KCl respectively. The total mass exchanged during the electrochemical process is in the order of 65–88, 40–56 and 50–60 g mol^−1^ for Li^+^, Na^+^, and K^+^ systems, respectively. Further, the water exchange trend in Cl^–^ systems is depicted for G‐CN (Figure 6C) and G–COOH (Figure 6D) electrode systems.

### 2.5 Molecular Dynamics Simulations

**Figure 7:**
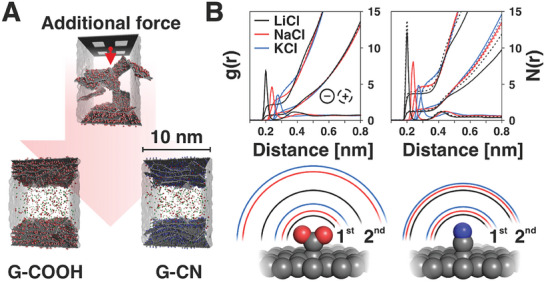
A) Scheme showing the electrode preparation procedure using the perforated flake and assembled cells (bottom) used in MD simulations. B) Radial distribution function, g(r), between individual cations for both negative and positive charged electrodes of G–COOH (left) and G–CN (right) and O atom of H_2_O molecules and associated cumulative number N(r). The boundaries of individual solvation shells of studied cations are graphically shown in the lower part.

In order to have further insight into the observed phenomena, we performed classical molecular dynamics (MD) simulations. We used the fixed charge method (FCM) to simulate graphene derivative (G‐CN/G‐COOH) based electrode charging. The complex electrode structures ordered into a lamellar morphology retained its stacking arrangement during MD simulations of both uncharged (resting) and charged states (see **Figure** **7**A). The layered structures of the used materials at electrodes were confirmed also by SEM images of the samples (Figure S1, Supporting Information). The formed layered slabs were ≈3.1 and 4.0 nm thick consisting of four distinct stacked layers of G–CN or G–COOH flakes, respectively. The interlayer distances ranged from 0.63 to 0.88 nm for G–CN and G–COOH, respectively. The increased value in the case of G–COOH was caused by the intercalated counter‐cations due to its negative surface charge (COO^−^) and associated water molecules. The prepared electrodes differed in their roughness. The solvent accessible surface areas (SASA) of G–CN and G–COOH reached 749.2 and 895.8 nm^2^, respectively.

We analyzed the behavior of aqueous 0.5 m KCl, NaCl, and LiCl electrolytes confined between a pair of electrodes in detail. We primarily focused on the interaction of ions with the electrodes and compared the uncharged and charged states. The most significant changes in water/ions organization were observed in the proximity of functional groups (Figure 7B). Since for the G–COOH on average 44–48% of all ions were present on the interface (considered all ions up to 0.5 nm from the surface) (see **Table** **1**). The differences between the individual ions were rather marginal, however, the biggest interface/bulk ratio was found for Li^+^ for both materials. Additionally, the biggest differences (in absolute values) between the adsorbed cations on positively and negatively charged electrode was observed for G‐COOH. However, the redistribution of charged species caused by the difference of electrochemical potential was more significantly pronounced in G–CN in comparison to G‐COOH (changes to the ratio to the total number of ions). The dissociated COO^–^ groups on G‐COOH governed the structuring of ions and the response on the external field was then less prominent. The density of chlorine anions was the same regardless of the electrolyte cation. A larger fraction of Cl^–^ resided at positively charged electrode balancing its opposite charge. Additionally, our results suggested that there are almost no differences in the density profiles of water regardless of the electrolyte (see Figure S21, Supporting Information). One can see the differences between the positively and negatively charged electrode, however, the type of cation has no effect on the shape of the curve. Moreover, the typical distinct two‐layered arrangement of water molecules that is usually visible near graphitic surfaces^[^ ^80^, ^81^ ^]^ was not formed. This is based on several facts: i) the surface functionalization disrupts the amount and the size of pure aromatic sp^2^ islands; and ii) despite the lamellar structure of the electrodes the surface complexity (edges, warped structure, small fragments) considerably hardens the water organization around the surface.

**Table 1:** Calculated number of exchanged water molecules and cations from MD simulations for G–CN and G–COOH material.

Number of exchanged	KCl		NaCl		LiCl	
	Water molecules	Cations	Water molecules	Cations	Water molecules	Cations
G‐CN	46.8	14.5	8.1	17.3	28.3	21.9
G‐COOH	155.6	89.9	102.1	70.1	182.6	37.5

Finally, we have evaluated changes of the water organization and its variation induced by an additional potential. Water formed a dense shell around the material (measured 0.5 nm from the surface of all of the individual sheets) containing 12% and 20% of all waters in the system for G–CN and G–COOH, respectively. The exceptional water‐rich environment around G–COOH was also reflected in the larger interlayer distance and could be associated with greater surface polarity, thus corroborating the experimental hypothesis. During the charging/discharging process, the greatest exchange of water molecules occurred in G–COOH as shown in Table 1.

Moreover, most waters were exchanged in the case of LiCl, despite the fact that the exchange of Li^+^ was the least profound. This might indicate that not all the exchanged water molecules are associated with a direct change in the solvation layer of individual Li^+^ cations. It can be explained by more demanding dehydration of smaller cations.^[^ ^82^ ^]^ They like to retain their solvation shell, resulting in less frequent interactions with the interphase.

## 3 Conclusion

Systematic and novel approaches bypass the limitations of synthesizing graphene derivatives under harsh conditions, yielding densely and selectively functionalized electrode materials. Graphene derivatives, such as G–F–CN, G–CN, G–COOH, G–COOH (O), G–N were investigated in charge storage applications toward univalent alkali metal cations of Li^+^, Na^+^, and K^+^. Systematic understanding of ion storage in these covalent derivatives, the influence of functionality, solvent exchange and/or assisted transfer during the electrochemical process, is vital for fabricating active and novel electrode materials for energy storage applications. Covalent functionalization of these graphene derivatives influenced the electrochemistry of materials and EQCM aided in giving information on the mass exchange of both cations and water molecules during the electrochemical process. MD simulations revealed that the complexity of the surface had a significant impact on the organization of the electrode/electrolyte interphase, both directly and indirectly through water structuring. Additionally, the MD simulations provided valuable insights into the behavior of the electrolyte/electrode interface during electrode charging. G–COOH (O) displayed high charge storage capability over other covalently functionalized derivatives, owing to the densely carboxylated conductive graphene surfaces and enhanced ─O‐terminated groups. The water molecules accompanying univalent alkali metal cations during the electrochemical process were analyzed, showing dependence on scan rates. We demonstrated here that dense functionalization of graphene with appropriate functional groups can strongly enhance their capability to incorporate alkali metal ions for energy applications. These findings shall have profound impact on the construction of future energy storage devices.

## 4 Experimental Section

### Materials and Characterization Techniques

Sodium cyanide (p.a. ≥ 97%), and graphite fluoride (>61 wt.% F, C_1_F_1.1_) were obtained from Merck. Acetone (pure), and ethanol (absolute) were purchased from Penta. Amine‐free dimethylformamide and nitric acid (Analpure, 65%) were obtained from Lach‐Ner. All aqueous solutions were prepared with ultrapure water (18 MΩ cm^−1^). Lithium sulfate, sodium sulfate, potassium sulfate, lithium chloride, sodium chloride, and potassium chloride were procured from Sigma Aldrich. Surface morphology and EDX mapping of synthesized graphene derivatives was studied using an SEM (TESCAN LYRA 3). Chemical compositional analyses were performed by XPS (Kratos AXIS Supra instrument) using a monochromatic Al Kα (1486.7 eV) excitation source. The X‐ray power was 225 W. The data were analyzed using Casa XPS software. Raman spectroscopy measurements were carried out using Alpha 300R instrument (WITEC, Germany) equipped with a CCD detector using 100× magnification, 15 mW laser power at 532 nm excitation wavelength. FTIR spectra were recorded on an iS5 FTIR spectrometer (Thermo Nicolet) using the Smart Orbit ZnSe ATR accessory. Briefly, a droplet of ethanol dispersion of the relevant material was placed on the ZnSe crystal and dried. The spectra were then acquired by summing 52 scans while using a nitrogen gas flow through the ATR accessory. ATR and baseline correction were applied to the collected spectra. Water contact angle measurements were carried out using the See System E Advex Instruments (SEE‐SYSTEM). Surface area and pore size analysis was performed by means of N_2_ adsorption/desorption measurements at 77 K on a volumetric gas adsorption analyzer (Autosorb iQ XR, Anton‐Paar Quanta Tec, USA) up to 0.965. Prior to the analysis, the sample was degassed under high vacuum (10–7 Pa) at 130 °C for 12 h, while high purity (99.999%) N_2_ and He gases were used for the measurements. The Brunauer–Emmett–Teller surface area (BET) was determined with respect to Rouquerol criteria for N_2_ isotherm. The pore size distribution was analyzed by the N_2_‐QSDFT 77‐carbon slit pore kernel.

### Synthesis of Functionalized Graphene Derivatives—Fluorine‐Doped Cyanographene (G–F–CN) and Cyanographene (G–CN)

Synthesis was carried out based on previously reported work.^[^ ^50^ ^]^ Initially, fluorinated graphite (120 mg, ≈4 mmol of C–F units) was added to DMF (15 mL) and sonicated (Bandelin Sonorex, DT 255H type, frequency 35 kHz, power 640 W, effective power 160 W) for 4 h under nitrogen atmosphere in a 25 mL round‐bottomed glass flask. Further, NaCN (800 mg; ≈16 mmol) was added to the above mixture and heated (130 °C) with a condenser, stirring at a speed of 500 rpm. Sample aliquots were removed at regular intervals from the flask to monitor the reaction progress with FTIR (see Figure 1a in ref. [50]). The product obtained after 1 h was used for subsequent experiments and this intermediate (termed here as G–F–CN) was cooled to room temperature (RT), after which an equal amount of acetone was added. Centrifugation was employed followed by washing steps in the order of DMF, dichloromethane, acetone, ethanol, and water (all 4×). Hot (80 °C) DMF and water were also used. The washing steps were increased using DMF and water if the conductivity of the supernatant aqueous portion was higher than 200 µS cm^−1^. A centrifugal force of up to 25000 rcf was employed in the final steps of centrifugation with water to isolate the product. G–F–CN was washed with acidified water (pH = 4) to exchange sodium cations (Na^+^) with H_3_O^+^. After washing, the procured material was suspended in absolute ethanol, pure DMF, or pure water depending on the application. Defluorinated cyanographene (G–CN) was obtained following the same procedure keeping a reaction time of 24 h. The functionalization degree of G–CN was 15%, as previously determined from XPS analysis.^[^ ^50^ ^]^

### Synthesis of Functionalized Graphene Derivatives—Graphene Acid (G–COOH)

G–COOH was synthesized according to the literature^[^ ^50^ ^]^ wherein G–CN was hydrolyzed with a nitric acid solution (HNO_3_; 20%). The mixture was heated (100 °C) under reflux with stirring (350 rpm) for 24 h. Further, the G–COOH was washed in successive separation/dispersion cycles and freeze–dried. The functionalization degree of G–COOH was 13%, as previously determined from XPS analysis.^[^ ^50^ ^]^

### Synthesis of Functionalized Graphene Derivatives—Oxidized Graphene Acid (G–COOH (O))

Oxidized graphene acid was obtained as previously described,^[^ ^53^ ^]^ where G–COOH was additionally treated in a 40% HNO_3_ solution, under reflux and stirring (500 rpm) for 24 h. The product was isolated and washed with H_2_O by centrifugation. When it stopped precipitating after a few washing steps, acidic water (pH = 4) was used to protonate the material and reduce its dispersibility, inducing precipitation. Finally, dialysis was applied to obtain stable aqueous suspensions of G–COOH (O) with a final pH of ≈3.2 and a suspension conductivity of ≈150 µS cm^−1^.

### Synthesis of Functionalized Graphene Derivatives—Nitrogen‐Superdoped Graphene (G‐N)

G–N synthesis was adopted from a previous publication.^[^ ^54^ ^]^ 1 g of graphite fluoride was dispersed in DMF (in 60 mL) and stirred for three days at 500 rpm under a nitrogen atmosphere in a glass flask. Further, the procured mixture was sonicated for 4 h using Bandelin Sonorex, DT255H type, frequency 35 kHz, with an effective power 160 W, and then left for stirring overnight. Further, 3 g of NaN_3_ was added and the dispersion was heated (130 °C for 72 h) with a condenser and stirred at 800 rpm in an oil bath. The mixture was cooled down and washed successively using DMF, acetone, ethanol, hot water (80 °C), water, and acidified water (3% solution of HCl). The samples were centrifuged further at 13 000 rcf to separate products from the solvent. Later, the samples were washed with water until the active material stopped precipitating with a centrifuge. The dispersion was finally subjected to dialysis against ultrapure water, until the conductivity of the dispersion was in the order of 0.2 mS cm^−1^ or below.

### Sample Preparation and EQCM Measurements

Initially, aqueous dispersion of 1 mg mL^−1^ of sample was sonicated along with nafion binder (9:1) for 30 min. The uniformly dispersed samples were later spray‐coated over an Au quartz crystal and dried prior to measurement. The graphene derivatives were loaded over an Au quartz resonator that served as the working electrode, with Ag/AgCl and platinum as the reference and counter electrode, respectively. The potential window was confined between 0.4 and −0.4 V versus Ag/AgCl during measurement. Prior to electrochemical measurements, the electrode was cycled ten times to obtain stable CV measurements in 0.5 m solutions of Li_2_SO_4_, Na_2_SO_4_, K_2_SO_4,_ LiCl, NaCl, and KCl. The mass change (Δm) of the active material that is loaded on the quartz crystal during the electrochemical process can be determined by the microbalance frequency change (Δf) through the Sauerbrey equation.^[^ ^62^ ^]^

### Simulation Setup

Cyanographene (G–CN) and graphene acid (G–COOH) flakes were prepared by both side functionalization of graphene carbon honeycomb lattice by nitrile and carboxyl groups, respectively, using 13% degree of functionalization in accord with experiments.^[^ ^50^ ^]^ The initial structures of G–CN or G–COOH based electrodes were prepared by random arrangement of different sizes and shapes of functionalized graphene flakes (5 pcs. of rectangular flakes each ≈4 × 4 nm, 5 pcs. of ≈3 × 6 nm, and 5 pcs. of ≈2 × 4 nm and 5 circumcoronenes) over a periodic G–CN/G–COOH sheet (10.1 × 10.2 nm). The system was solvated, minimized, thermalized (10–300 K; 5 ns) and let evolve over 40 ns. The resulting self‐assembled structure was gradually compressed (for a total time of 150 ns) by a perforated (enabling water to escape from the compressed areas) graphene sheet piston that was pulled using a rigid constraint between reference groups toward the periodic sheet (cf. Figure 7A). After the compression the graphene piston flake was removed, and the system was additionally relaxed for 10 and 40 ns in the NpT and NVT ensemble, respectively. The obtained lamellar structures were then used for the assembly of an electrochemical cell, where they were placed at a distance of ≈8 nm representing the negative and positive electrode and solvated again with the corresponding electrolyte (0.5 m KCl, NaCl, or LiCl). Alkali metal chlorides are well‐parametrized and validated monovalent ion models used in molecular dynamics (MD) simulations across many fields of research.^[^ ^83^, ^84^ ^]^ Each cell was equilibrated for 5 ns in the NPT ensemble. After the slab width equilibration, the z‐coordinate in the final stage was extended to 3.5 times the length of the box, and the outer periodic sheets were restrained (the outside of the electrode was surrounded by a vacuum). The prepared cell was finally simulated for 100 ns in the NVT ensemble (the last 80 ns were considered for the final analysis). Partial charges of COOH/COO^−^ and CN groups were derived using the RESP method on a functionalized pyrene.^[^ ^85^ ^]^ Simulating pH neutral systems, the G–COOH molecule was modeled in its deprotonated form based on its pKa value of 5.3,^[^ ^50^ ^],^ i.e., all groups that were in contact with water (closer than 0.3 nm; in total 63% of all COOH groups) were deprotonated (this arrangement was then kept constant during the simulation). The resulting charge was neutralized by the corresponding countercations. The charging of the electrode was modeled using the fixed charge method (FCM) by placing ±0.002 e on each electrode atom depending on the corresponding electrode (mimicking the electric potential difference after electrode charging). Periodic boundary conditions were used in all three dimensions.

All simulations were performed in GROMACS 2019^[^ ^86^ ^]^ using the AMBER99 force field^[^ ^87^ ^]^ and SPC/E explicit water model.^[^ ^88^ ^]^ Graphitic carbons were modeled as uncharged LJ spheres using Cheng and Steele parameters.^[^ ^89^ ^]^ Electrolytes are simulated using Joung–Cheatham ion parameters.^[^ ^90^ ^]^ The equations of motion were integrated with a 2‐fs time step. The cut‐off for non‐bonded interactions was set to 1 nm. Electrostatics was treated using Particle–Mesh Ewald method (PME) and the real space term was truncated at the same cut‐off. Unless otherwise specified, the temperature was coupled to the V‐rescale thermostat^[^ ^91^ ^]^ with a 0.1 ps coupling constant, and the pressure was maintained at the reference 1 bar using the semi‐isotropic Berendsen barostat^[^ ^92^ ^]^ and 1.0 ps coupling constant. Hydrogen atoms were constrained by the LINCS algorithm.^[^ ^93^ ^]^ Figures were generated using PyMOL.^[^ ^94^ ^]^

## Conflict of Interest

The authors declare no conflict of interest.

## Author Contributions

A.K.K.P. performed investigation, methodology, conceptualization, formal analysis, data curation, material characterizations, experimentation, catalyst preparation and electrochemical measurements, validation, and discussions, and acquired funding, and wrote the original draft, and reviewed and edited the final manuscript. M.P. and M.O. performed theoretical calculations, formal analysis, validation, discussions, and methodology, acquired funding, and wrote, reviewed, and edited the final manuscript. A.B. performed formal analysis, methodology, validation, discussions, FTIR characterization, acquired funding, and wrote, reviewed, and edited the final manuscript. R.Z. performed methodology, formal analysis, validation, and discussions, acquired funding, and wrote, reviewed, and edited the final manuscript. M.P. performed supervision, methodology, formal analysis, validation, and discussions, acquired funding, and wrote, reviewed, and edited the final manuscript.

## Supporting information

Supporting Information

## Data Availability

The data that support the findings of this study are available from the corresponding author upon reasonable request.
