# Supplementary material for: Real Time Tracking of Nanoconfined Water‐Assisted Ion Transfer in Functionalized Graphene Derivatives Supercapacitor Electrodes
Source: Adv Sci (Weinh). 2024 Aug 6;11(39):2307583. doi: 10.1002/advs.202307583 (PMC11497090; doi:10.1002/advs.202307583)
Supplement: Supplementary file 1 — Supporting Information [file ADVS-11-2307583-s001.pdf]

ADVANCED SCIENCE
Open Access

Supporting Information

for *Adv. Sci.*, DOI 10.1002/advs.202307583

Real Time Tracking of Nanoconfined Water-Assisted Ion Transfer in Functionalized Graphene Derivatives Supercapacitor Electrodes

*Akshay Kumar K. Padinjareveetil, Martin Pykal, Aristides Bakandritsos, Radek Zbořil, Michal Otyepka and Martin Pumera**

Real Time Tracking of Nanoconfined Water-Assisted Ion Transfer in Functionalized Graphene Derivatives Supercapacitor Electrodes

Akshay Kumar K. Padinjareveetil,[a] Martin Pykal,[b] Aristides Bakandritsos,[b,c] Radek Zbořil,[b,c] Michal Otyepka,[b,d], Martin Pumera[a,e,f,g*]

[a] Future Energy and Innovation Laboratory, Central European Institute of Technology, Brno University of Technology, Purkyňova 123, Brno 61200, Czech Republic

[b] Regional Centre of Advanced Technologies and Materials, Czech Advanced Technology and Research Institute (CATRIN), Palacký University Olomouc, Olomouc 783 71, Czech Republic

[c] Nanotechnology Centre, Centre for Energy and Environmental Technologies, VŠB–Technical University of Ostrava, 17. listopadu 2172/15, Ostrava-Poruba 708 00, Czech Republic

[d] IT4Innovations, VŠB–Technical University of Ostrava, 17. listopadu 2172/15, Ostrava-Poruba 708 00, Czech Republic

[e] Advanced Nanorobots & Multiscale Robotics Laboratory, Faculty of Electrical Engineering and Computer Science, VSB - Technical University of Ostrava, 17. listopadu 2172/15, Ostrava 708 00, Czech Republic

[f] Department of Chemical and Biomolecular Engineering, Yonsei University, 50 Yonsei-ro, Seodaemun-gu, Seoul 03722, South Korea

[g] Department of Medical Research, China Medical University Hospital, China Medical University, No. 91 Hsueh-Shih Road, Taichung 40402, Taiwan

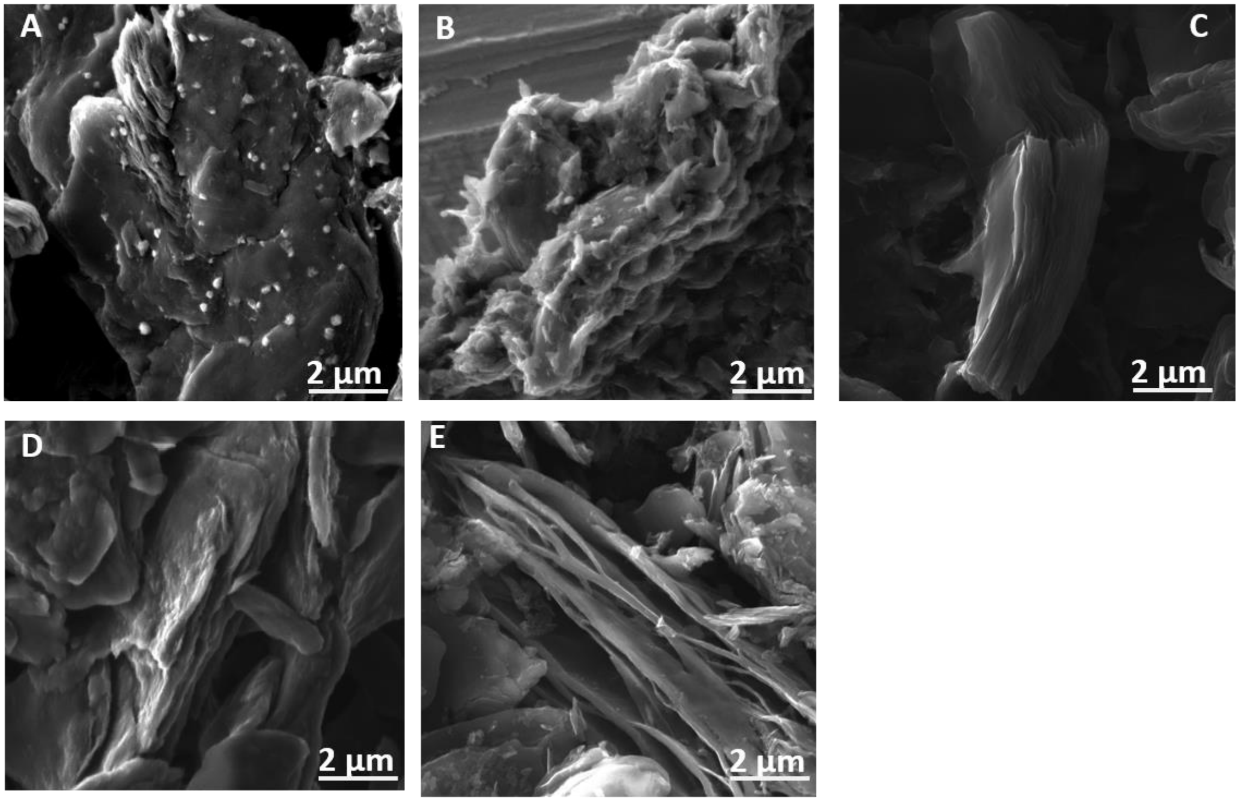

Figure S1. Scanning electron microscopy images of studied graphene derivative samples of A) fluorine-doped cyanographene (G-F-CN), B) cyanographene (G-CN), C) graphene acid (G-COOH), D) oxidized graphene acid (G-COOH (O)), and E) nitrogen superdoped graphene (G-N).

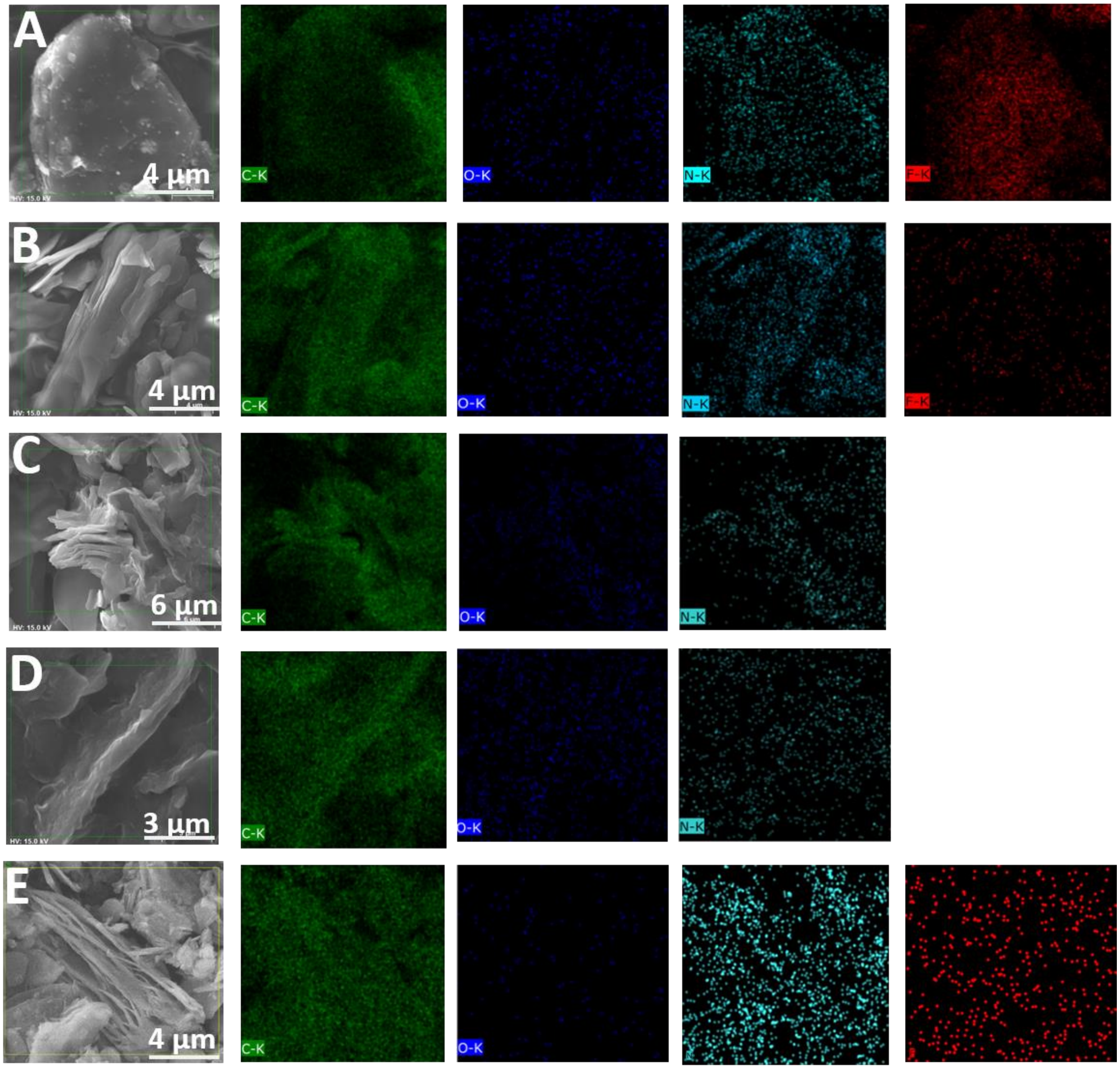

Figure S2. SEM and EDX mapping of A) fluorine-doped cyanographene (G-F-CN), B) cyanographene (G-CN), C) graphene acid (G-COOH), D) oxidized graphene acid (G-COOH (O)), and E) nitrogen superdoped graphene (G-N).

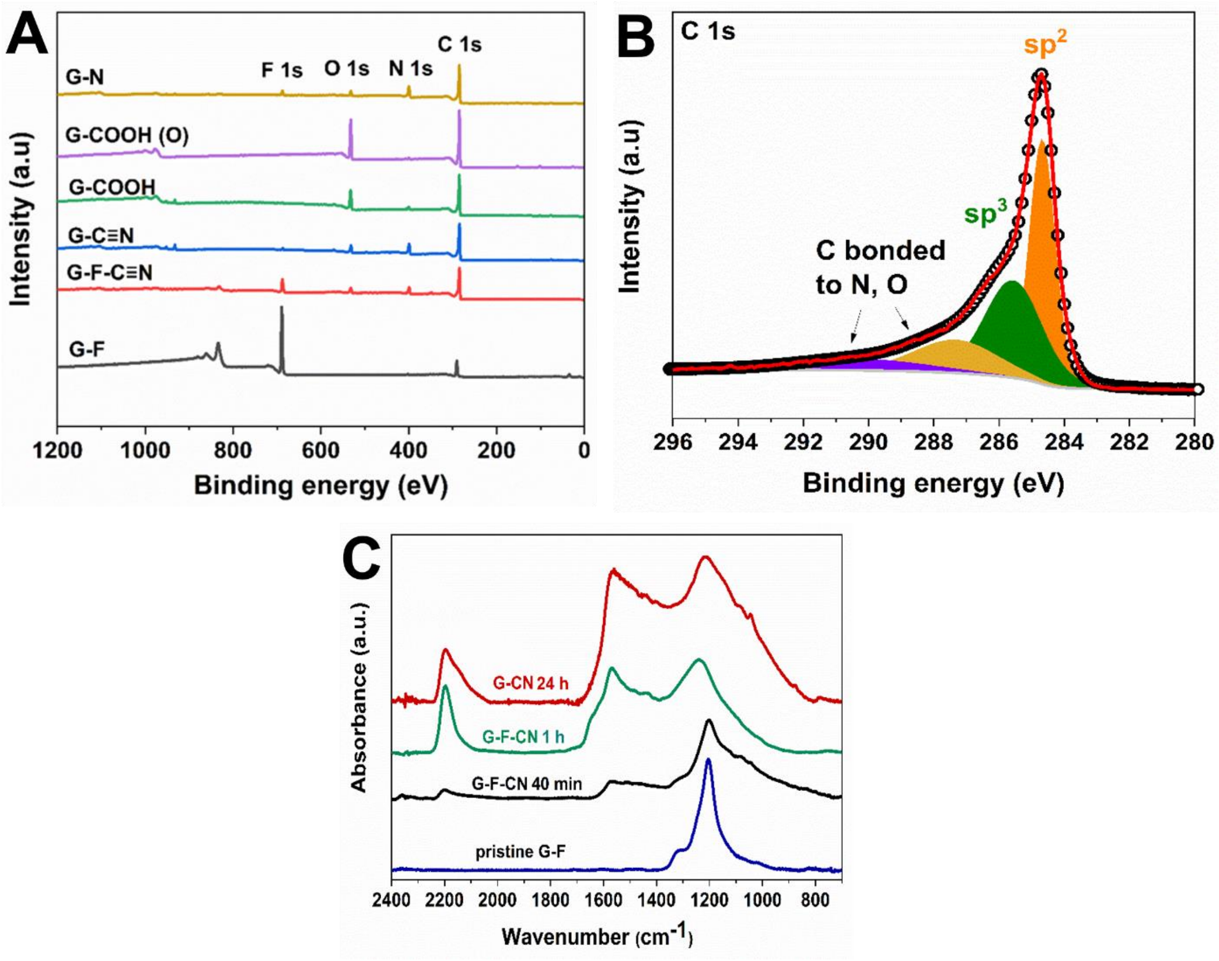

Figure S3. A) XPS survey spectra of graphene derivatives B) Deconvoluted C 1s spectra of G-CN samples. C) Fourier-transform infrared spectrum of fluorinated graphite (G-F), fluorine-doped cyanographene (G-F-CN) and cyanographene (G-CN). FTIR showed that the G-CN's spectrum was dominated by two bands at 1560 cm^{-1}, and between 1000 and 1210 cm^{-1}, both corresponding to skeletal vibrations of the sp^2 aromatic carbon network and to aromatic rings.[1,2] The same bands were present in F-doped G-F-CN derivative, but with lower relative intensity of the sp^2 bands, due to lower defluorination and aromatization. For comparison the G-F-CN sample withdrawn from the reaction at 40 min is also shown, where the nitrile and C=C bands are quite suppressed, and

only the band at 1200 cm^{-1} is dominant, where the C-F also appears. The spectra of G-F-CN 1 h and G-F-CN 40 min are reproduced from ref [50].

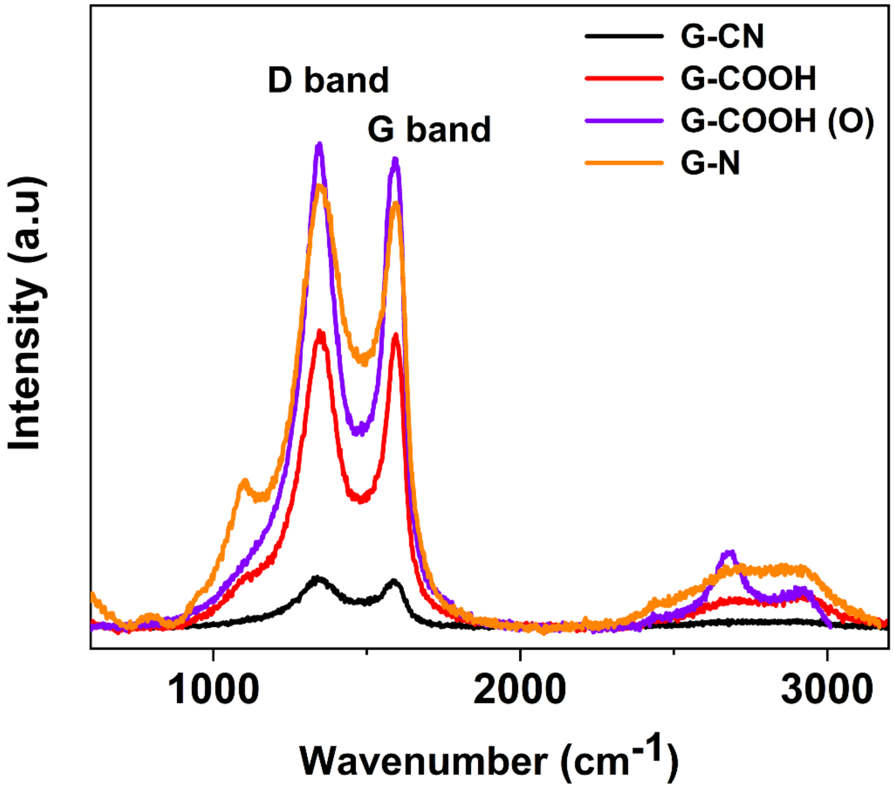

Figure S4. Raman signals of functionalized graphene derivatives. It is noted that FG does not have any Raman peaks due to the absence of aromatic rings and high fluorescence[3]. The band at low frequencies for the G-N product can be attributed to radial-like breathing mode vibrations, which is the relative in-plane vibration between the atoms close to the edges.[4] Due to the very high doping of G-N with pyrrolic and pyridinic nitrogen there are very high number of vacancies giving rise to many edge atoms. It is indicative that comparison of the Raman spectrum of G-N before and after annealing at 1000 °C (see [5] ref , Fig. S3 in that work), revealed the same Raman features

indicating that no healing process of the graphene backbone takes place during annealing and that the same amount and type of defects are preserved before and after the annealing. This corroborates the extensive holey structure observed from the TEM characterization, giving rise to permanent defects due to large vacancies. The I_D/I_G ratio of G-CN samples were around 1.05 and the broadening of bands are clear indications of highly functionalized graphene derivative. Upon comparing G-CN spectra with the G-COOH electrode the broadening of the bands is well evident, which accounts for the high functionalization. Further, upon evaluating the G-COOH (O), electrode material, a characteristic D band, G band, 2D band and D+D′ band were evident. Interestingly, a significant increase in the 2D band was evident in G-COOH (O) electrode material, upon comparison with G-COOH. Further, high degree of -O functionalization has resulted in the broadening of peaks in G-COOH (O) electrode materials, which also affirms the success of increased -O and -COOH functionalization.

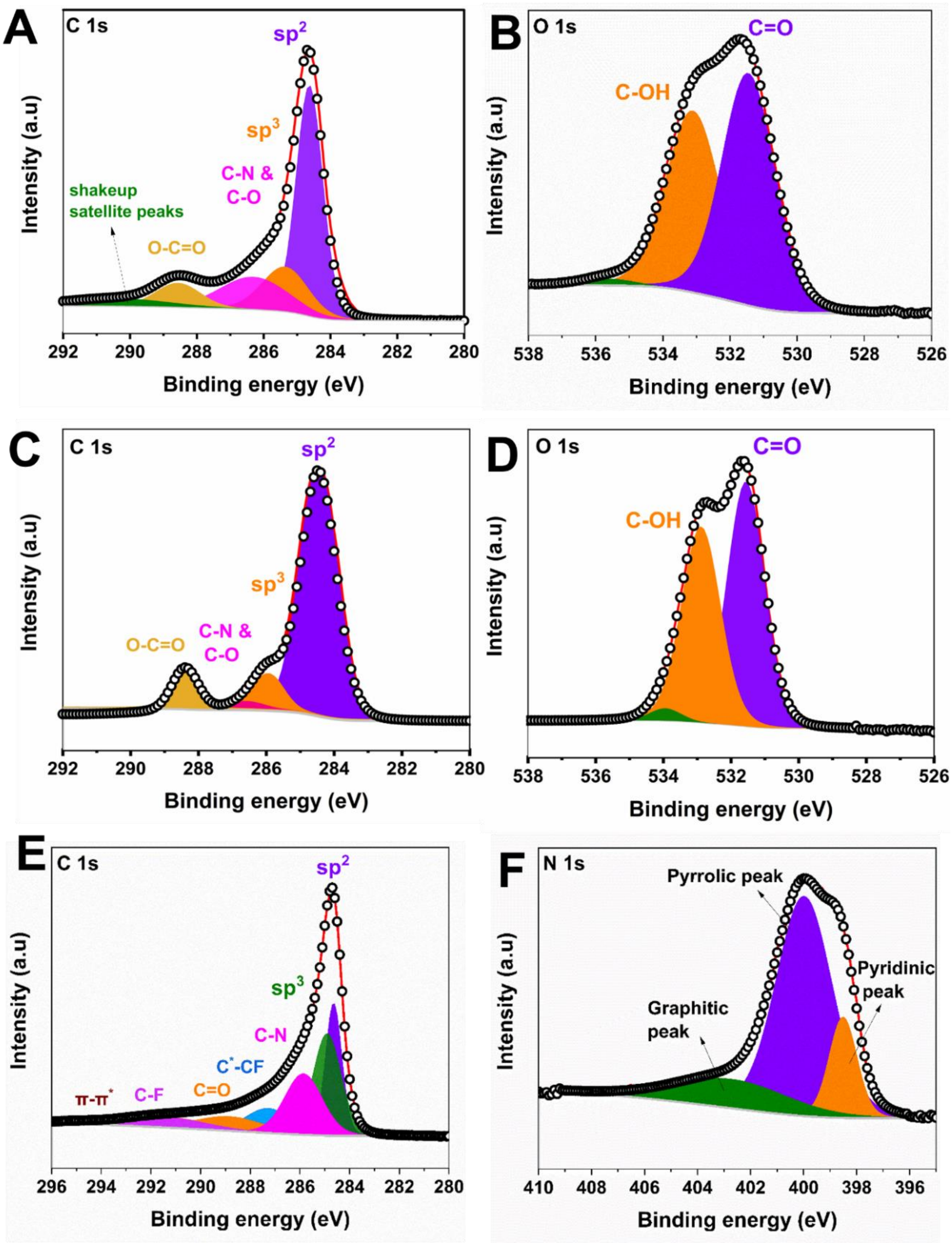

Figure S5. Deconvoluted C 1s and O 1s spectra of A, B) graphene acid; C, D) oxidized graphene acid respectively; Deconvoluted C 1s and N 1s spectra of E, F) nitrogen superdoped graphene respectively.

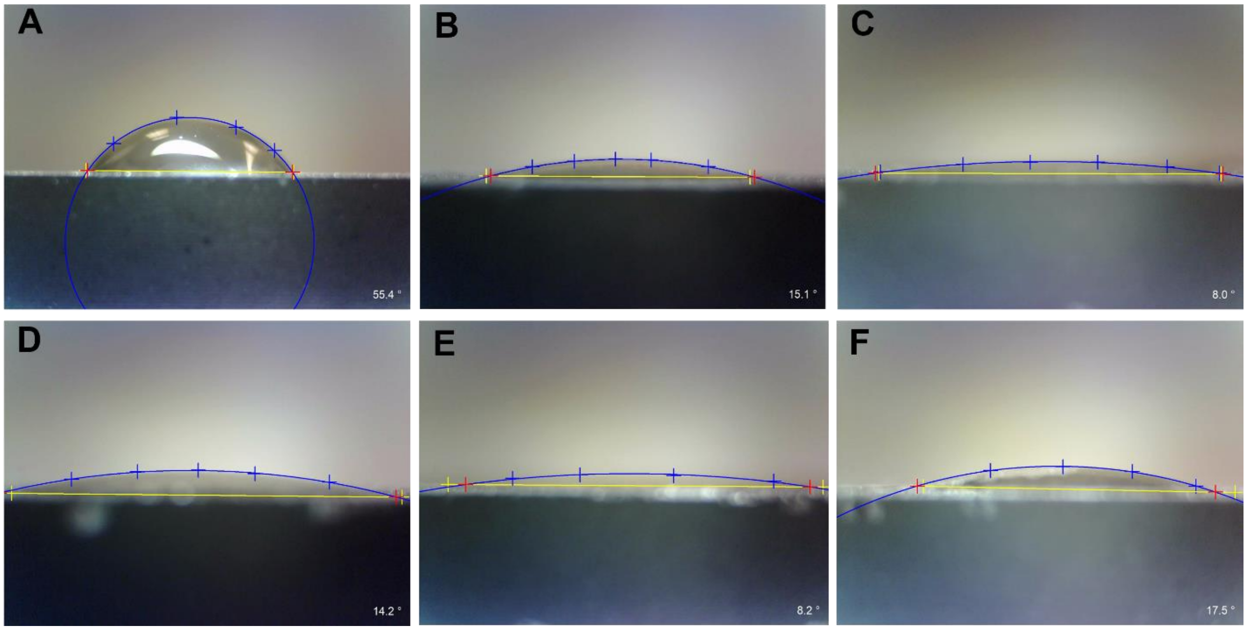

Figure S6. Water contact angle measurements of A) Si wafer substrate, B) G-F-CN, C) G-CN, D) G-COOH, E) G-COOH (O), F) G-N electrode material respectively.

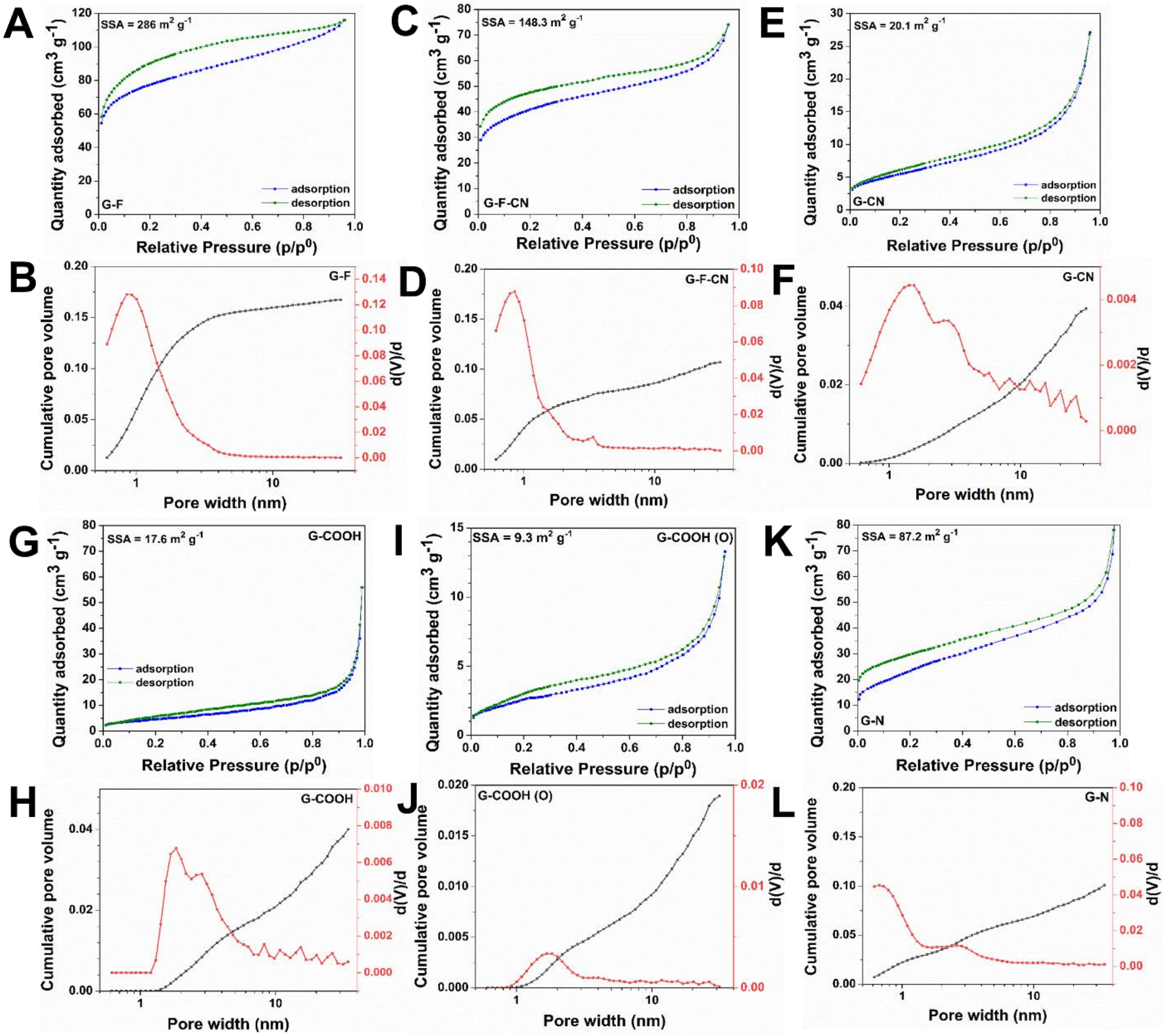

Figure S7. BET isotherm and pore size distribution plots of A, B) G-F; C, D) G-F-CN; E, F) G-CN; G, H) G-COOH; I, J) G-COOH (O); K, L) G-N electrode material respectively.

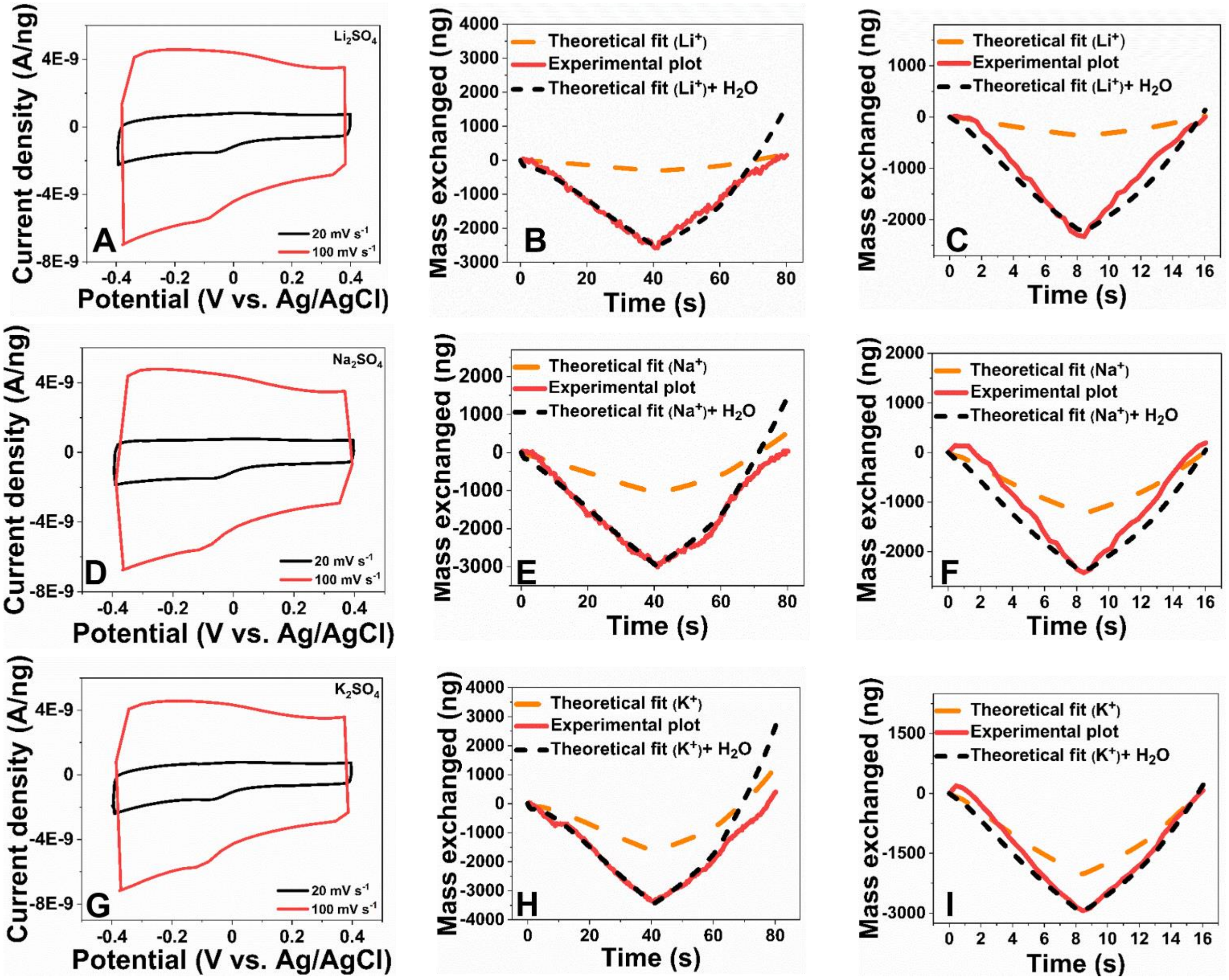

Figure S8. CV and mass exchange profile of G-F-CN electrode sample at 20 and 100 mV s^{-1} scan rates in A, B, C) 0.5 M Li_2SO_4; D, E, F) Na_2SO_4; G, H, I) K_2SO_4 respectively.

Table S1. Detailing on the ratio of cation to water exchange during the electrochemical process in various electrolytes procured from the mass exchange profile of each system.

	Graphene derivatives	Ratio	20 mV s^{-1}	50 mV s^{-1}	100 mV s^{-1}
T (a)	G-F-CN	Li^+/H_2O	2.77	2.11	2
		Na^+/H_2O	2.27	1.77	1.22
		K^+/H_2O	2.38	1.27	1
T (b)	G-CN	Li^+/H_2O	2.38	1.83	1.72
		Na^+/H_2O	1.88	1.33	1.22
		K^+/H_2O	1.38	0.77	0.27
T (c)	G-COOH	Li^+/H_2O	2.11	1.83	1.55
		Na^+/H_2O	1.38	1.05	0.67
		K^+/H_2O	1.72	0.88	0.78
T (d)	G-COOH (O)	Li^+/H_2O	8.94	8.22	8.21
		Na^+/H_2O	2.66	2	1.88
		K^+/H_2O	4.55	3.55	2.5
T (e)	G-N	Li^+/H_2O	2.27	1.5	1.33
		Na^+/H_2O	1.22	0.5	0.27
		K^+/H_2O	0.5	0	0

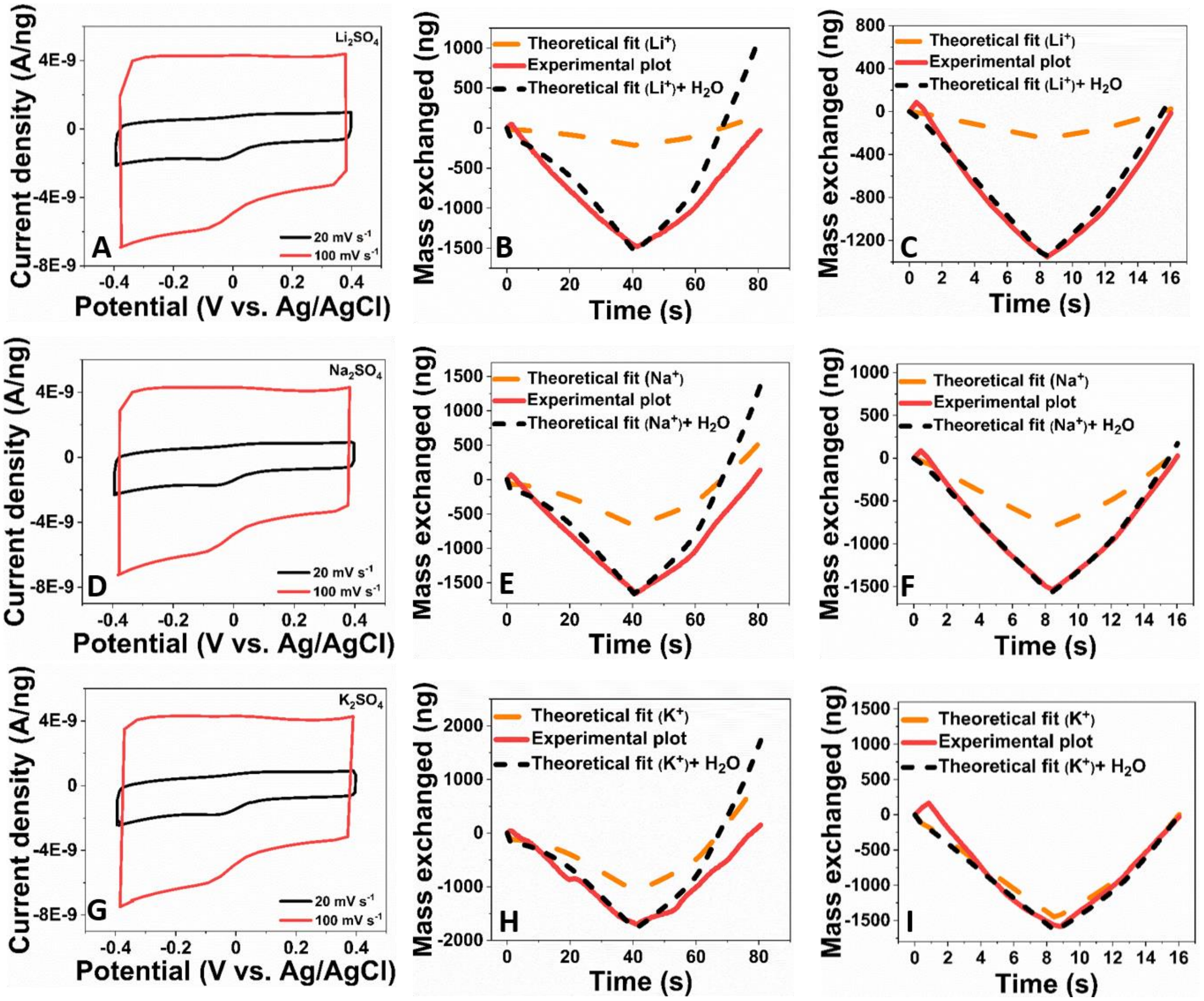

Figure S9. CV and mass exchange profile of G-CN electrode sample at 20 and 100 mV s^{-1} scan rates in A, B, C) 0.5 M Li_2SO_4; D, E, F) Na_2SO_4; G, H, I) K_2SO_4 respectively.

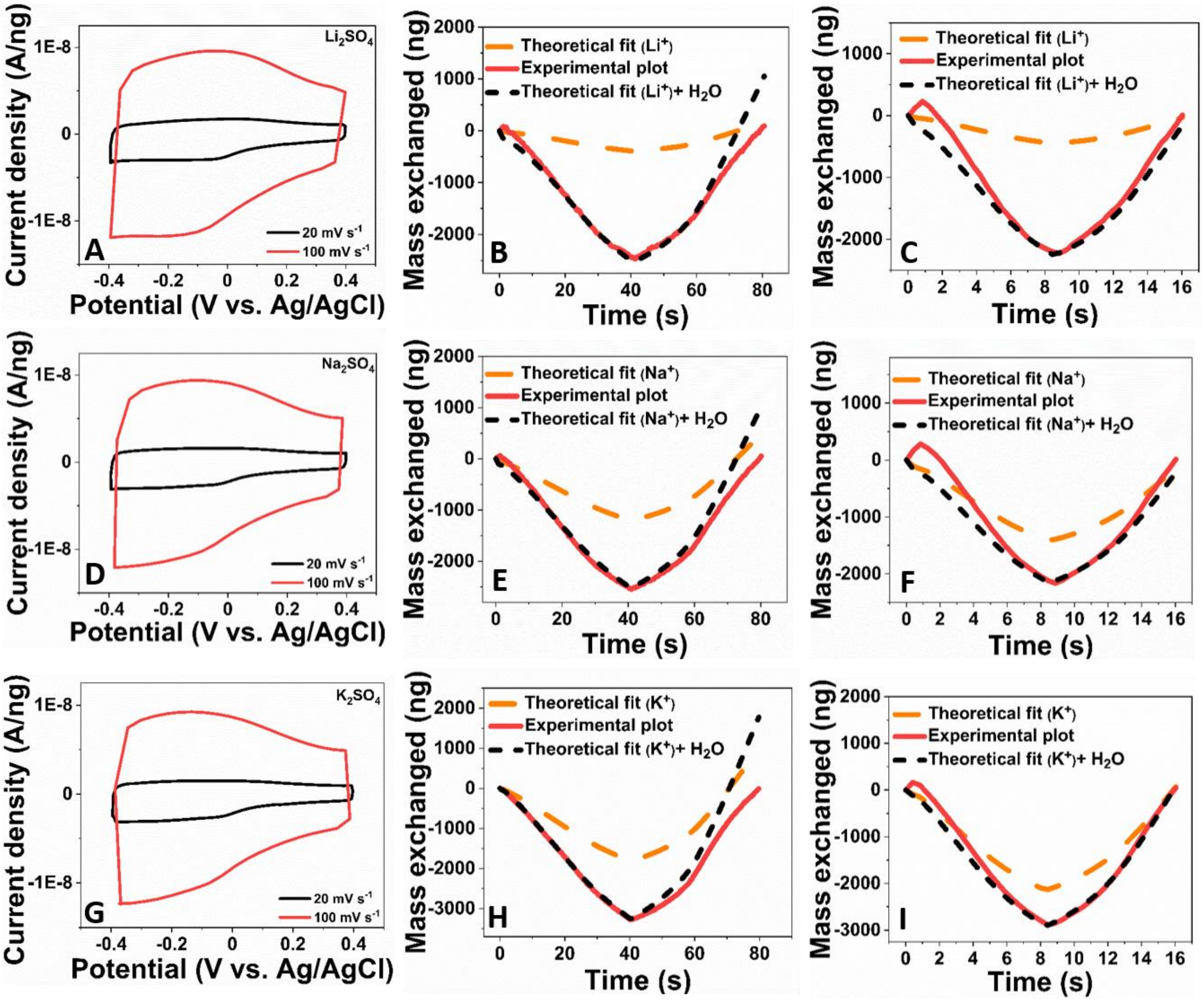

Figure S10. CV and mass exchange profile of G-COOH electrode sample at 20 and 100 mV s^{-1} scan rates in A, B, C) 0.5 M Li_2SO_4; D, E, F) Na_2SO_4; G, H, I) K_2SO_4 respectively.

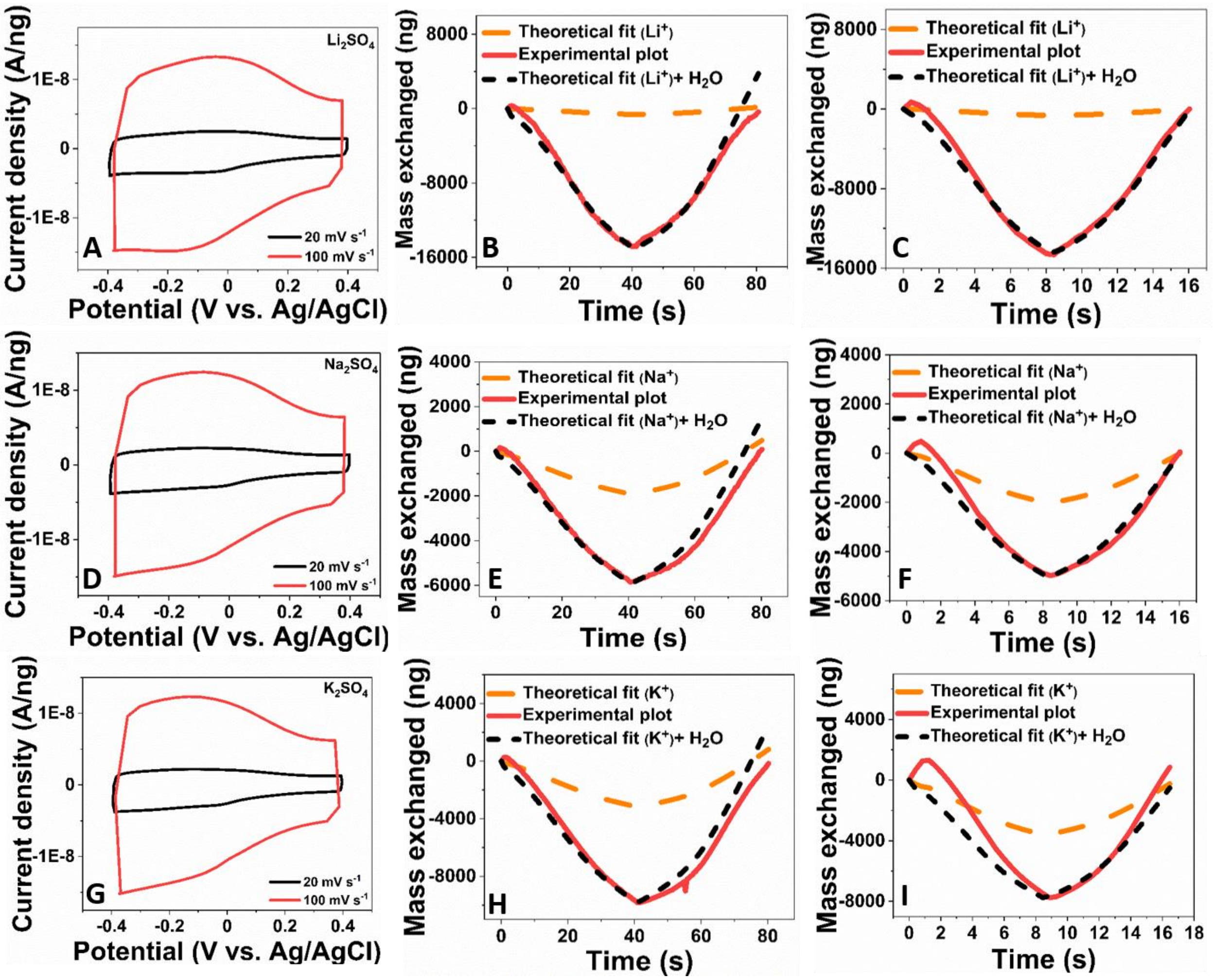

Figure S11. CV and mass exchange profile of G-COOH (O) electrode sample at 20 and 100 mV s^{-1} scan rates in A, B, C) 0.5 M Li_2SO_4; D, E, F) Na_2SO_4; G, H, I) K_2SO_4 respectively.

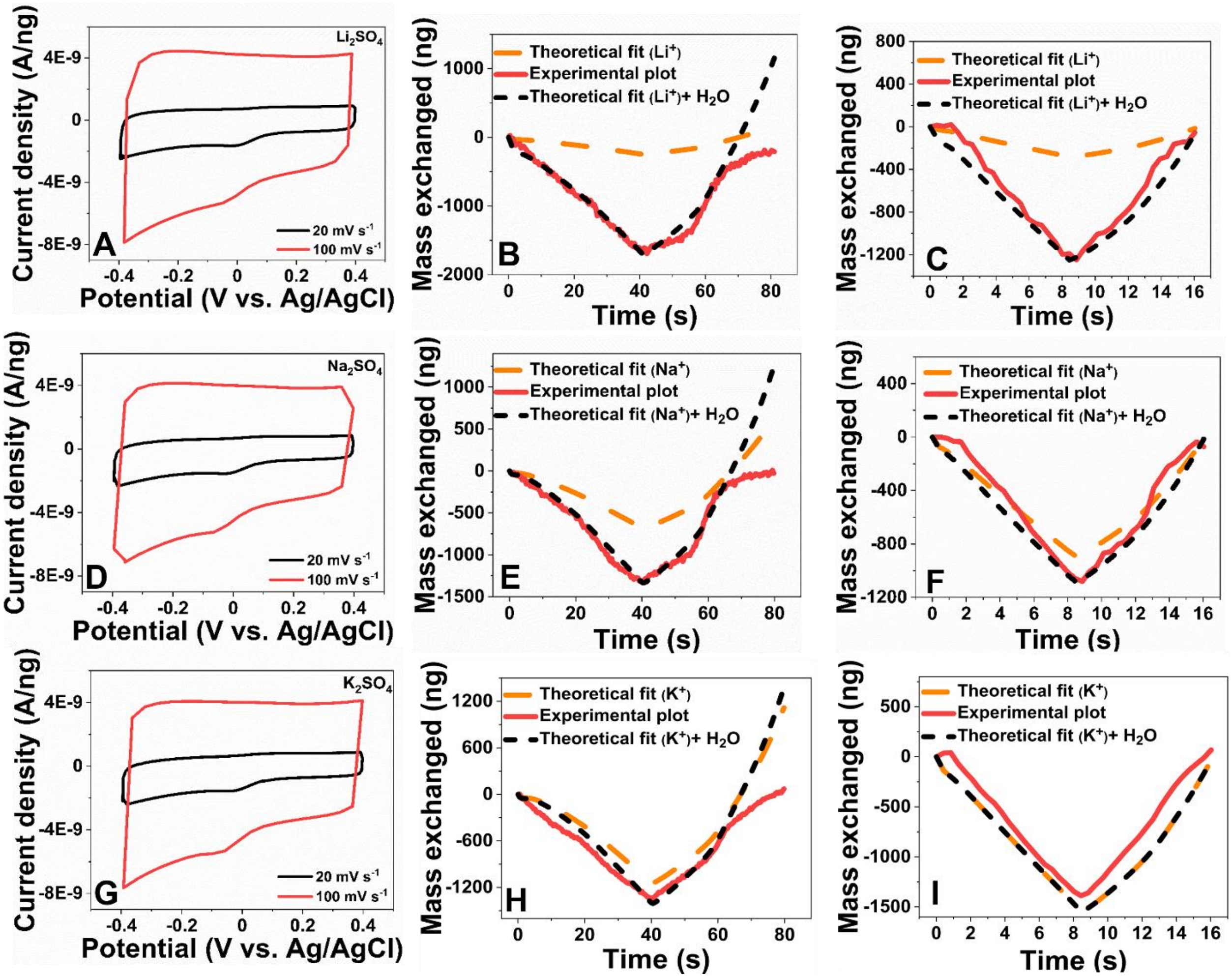

Figure S12. CV and mass exchange profile of G-N electrode sample at 20 and 100 mV s^{-1} scan rates in A, B, C) 0.5 M Li_2SO_4; D, E, F) Na_2SO_4; G, H, I) K_2SO_4 respectively.

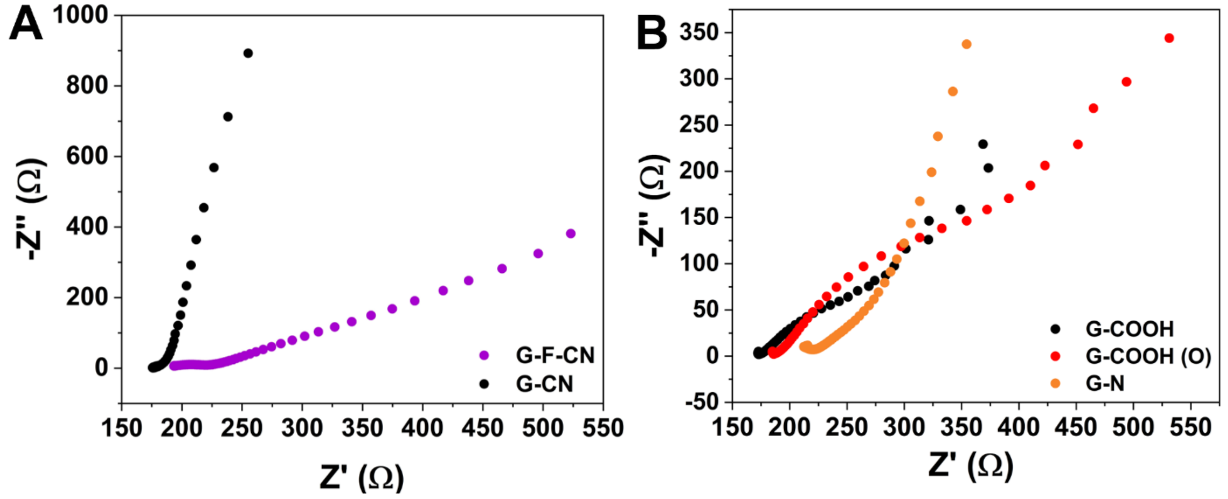

Figure S13. A, B) EIS measurements on graphene electrode materials.

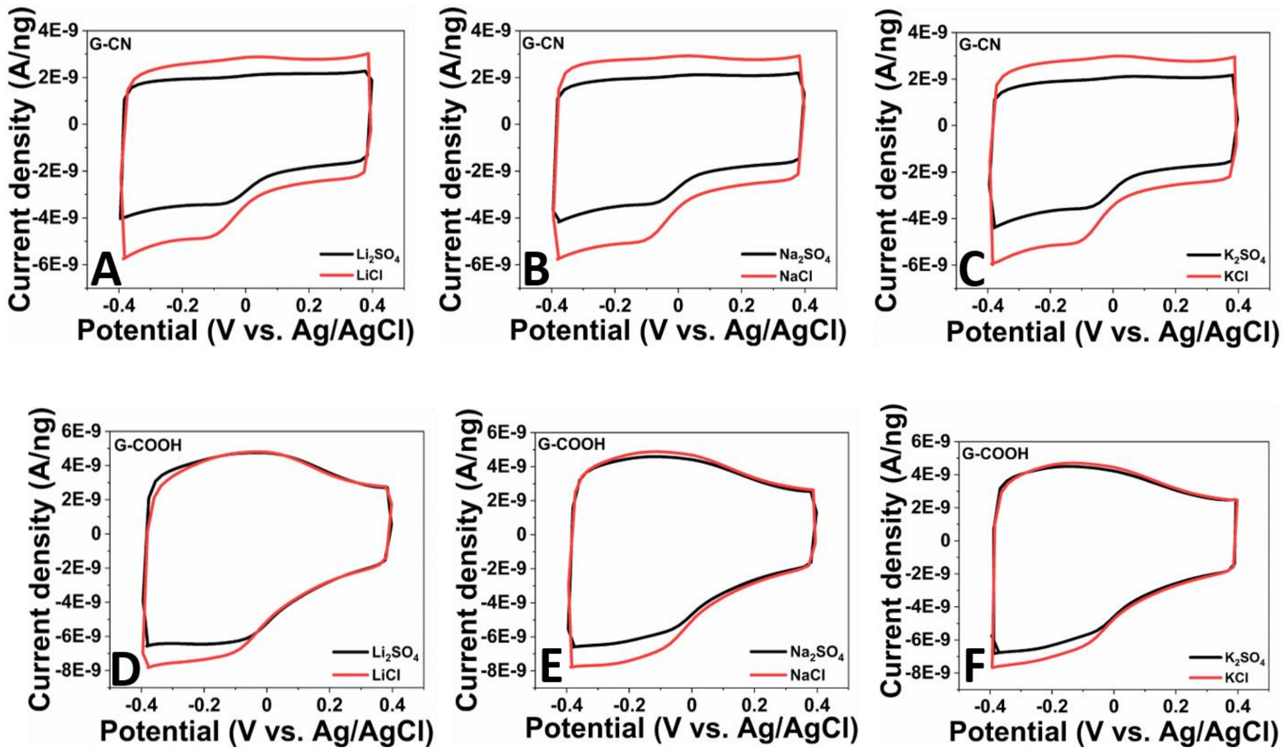

Figure S14. A-F) CV profile of graphene electrode material in various electrolyte solutions at 50 mV s^{-1}.

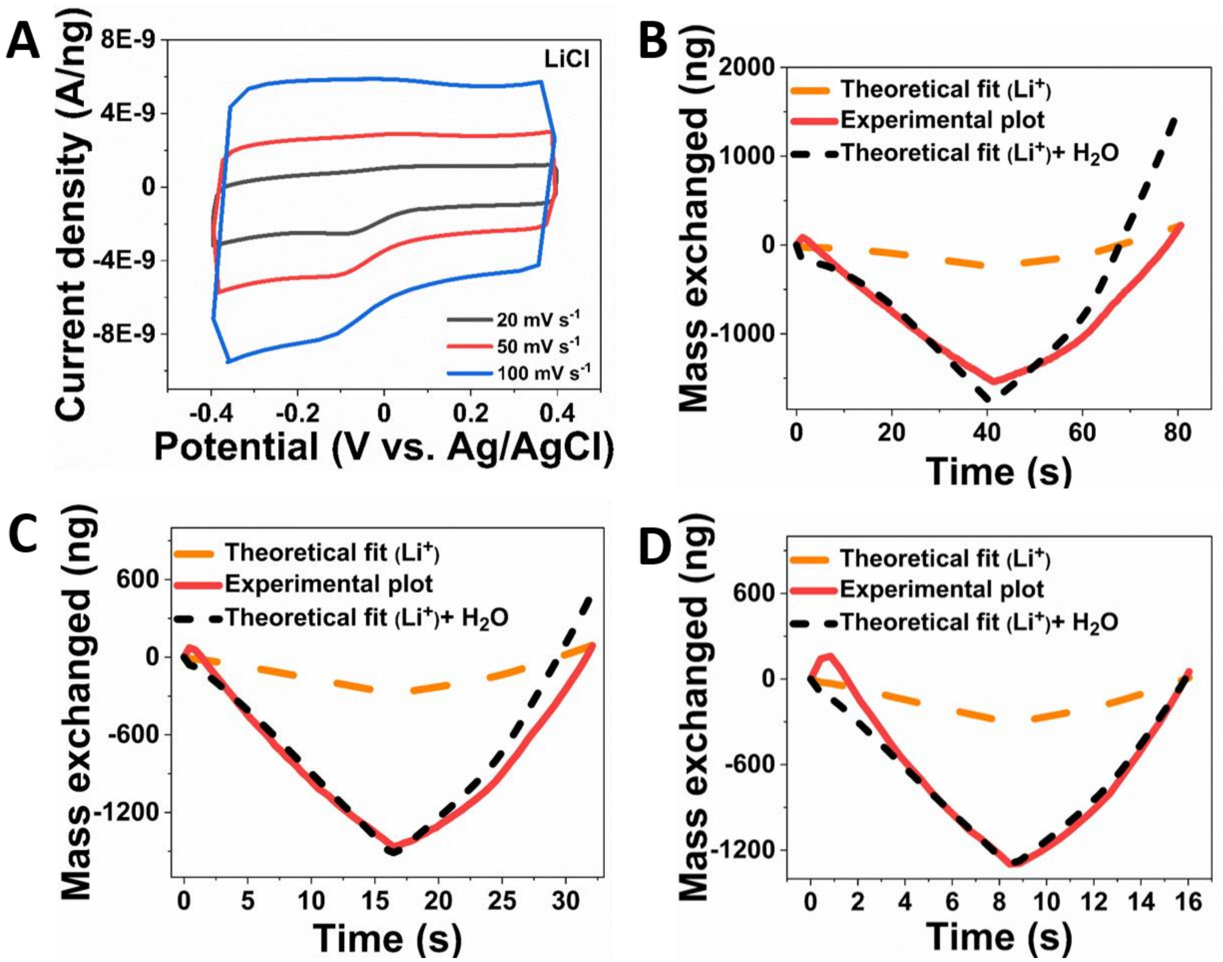

Figure S15. A) CV and mass exchange profile of G-CN electrode in LiCl electrolyte solution at scan rates of B) 20, C) 50, and D) 100 mV s^{-1}.

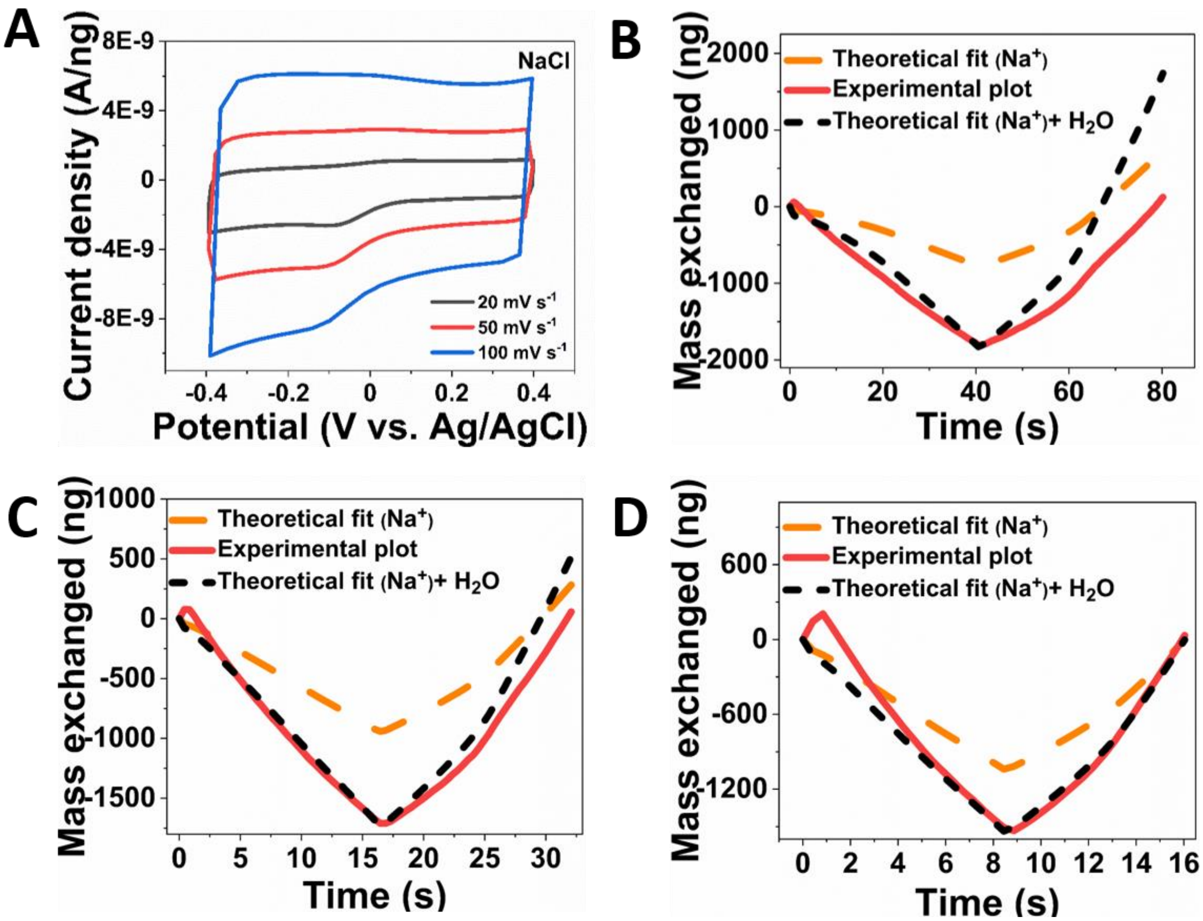

Figure S16. A) CV and mass exchange profile of G-CN electrode in NaCl electrolyte solution at scan rates of B) 20, C) 50, and D) 100 mV s^{-1}.

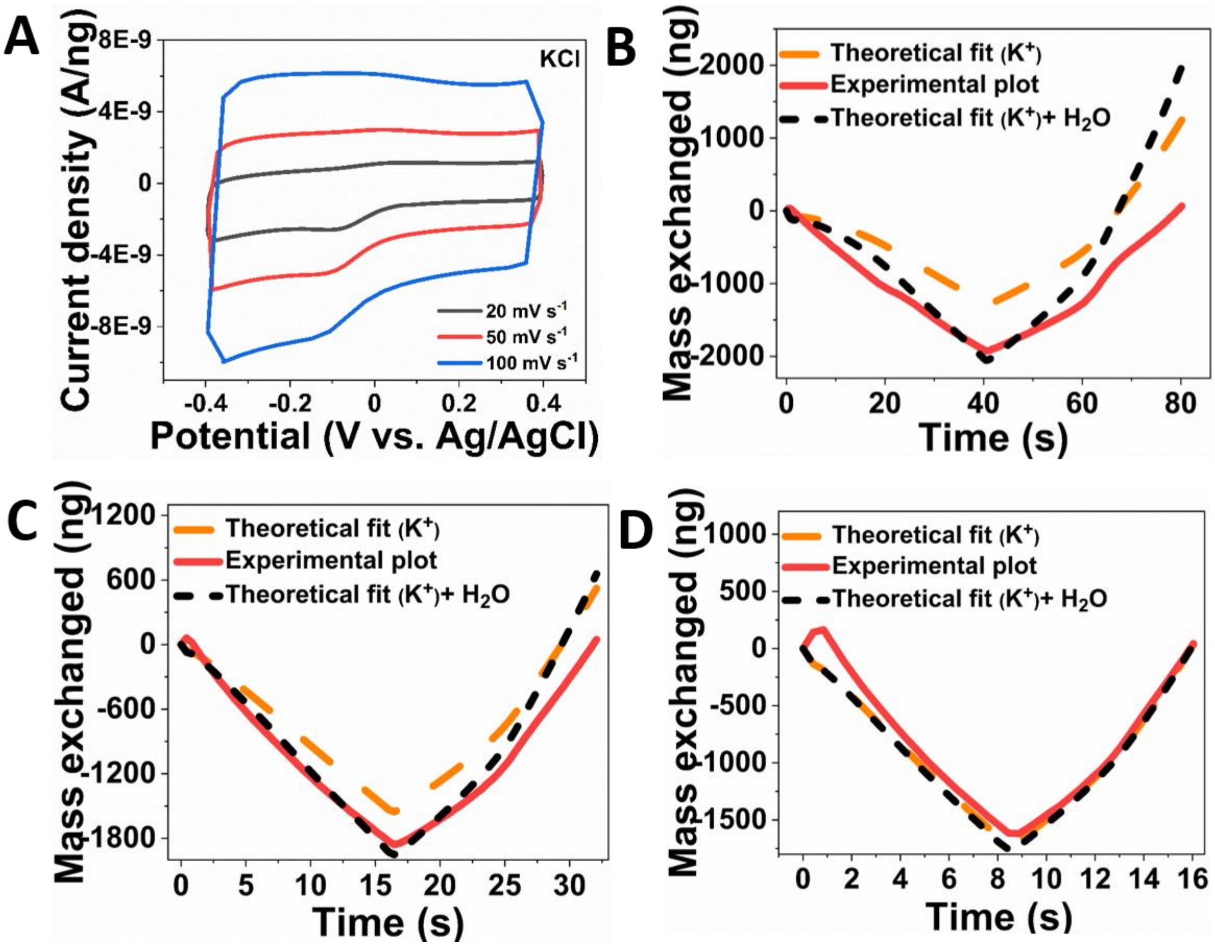

Figure S17. A) CV and mass exchange profile of G-CN electrode in KCl electrolyte solution at scan rates of B) 20, C) 50, and D) 100 mV s^{-1}.

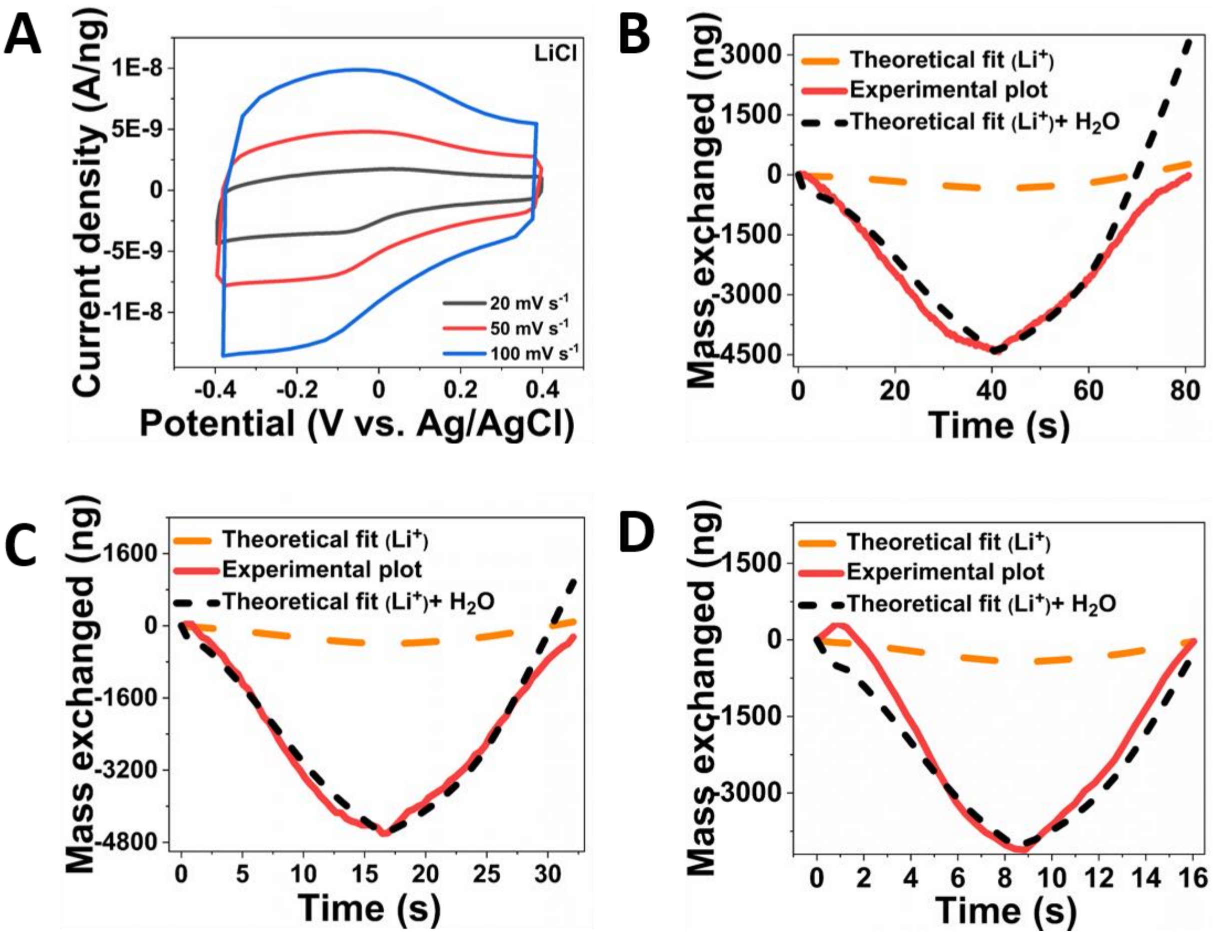

Figure S18. A) CV and mass exchange profile of G-COOH electrode in LiCl electrolyte solution at scan rates of B) 20, C) 50, and D) 100 mV s^{-1}.

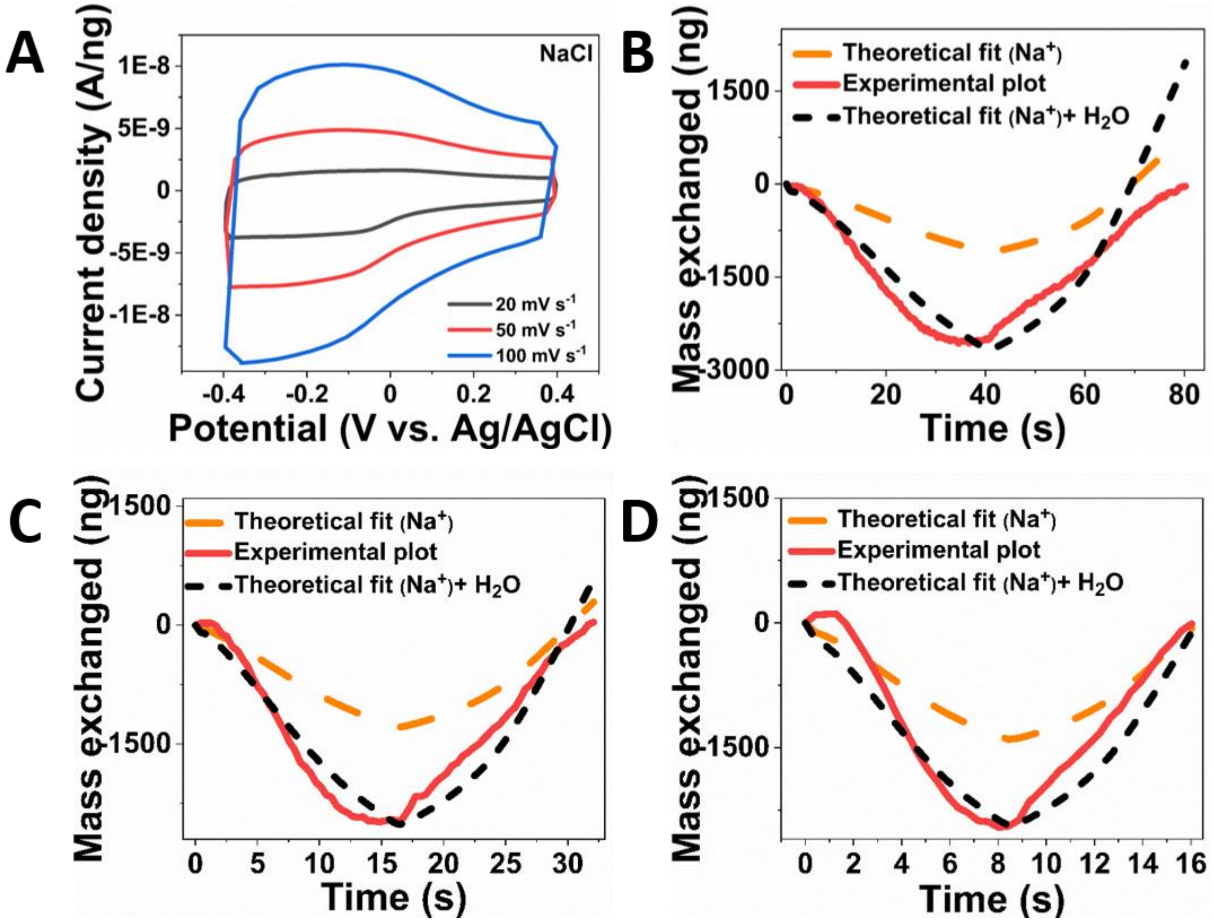

Figure S19. A) CV and mass exchange profile of G-COOH electrode in NaCl electrolyte solution at scan rates of B) 20, C) 50, and D) 100 mV s^{-1}.

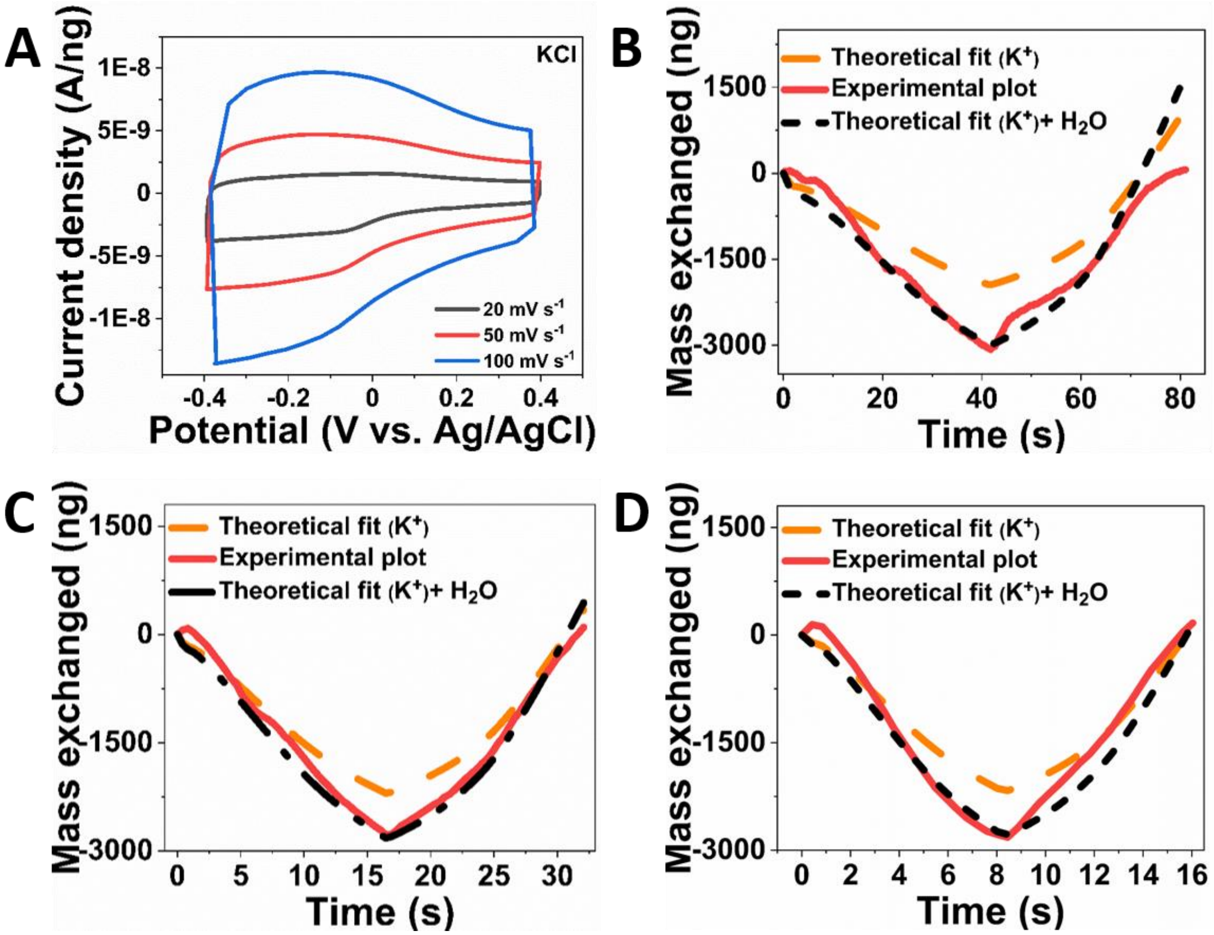

Figure S20. A) CV and mass exchange profile of G-COOH electrode in KCl electrolyte solution at scan rates of B) 20, C) 50, and D) 100 mV s^{-1}.

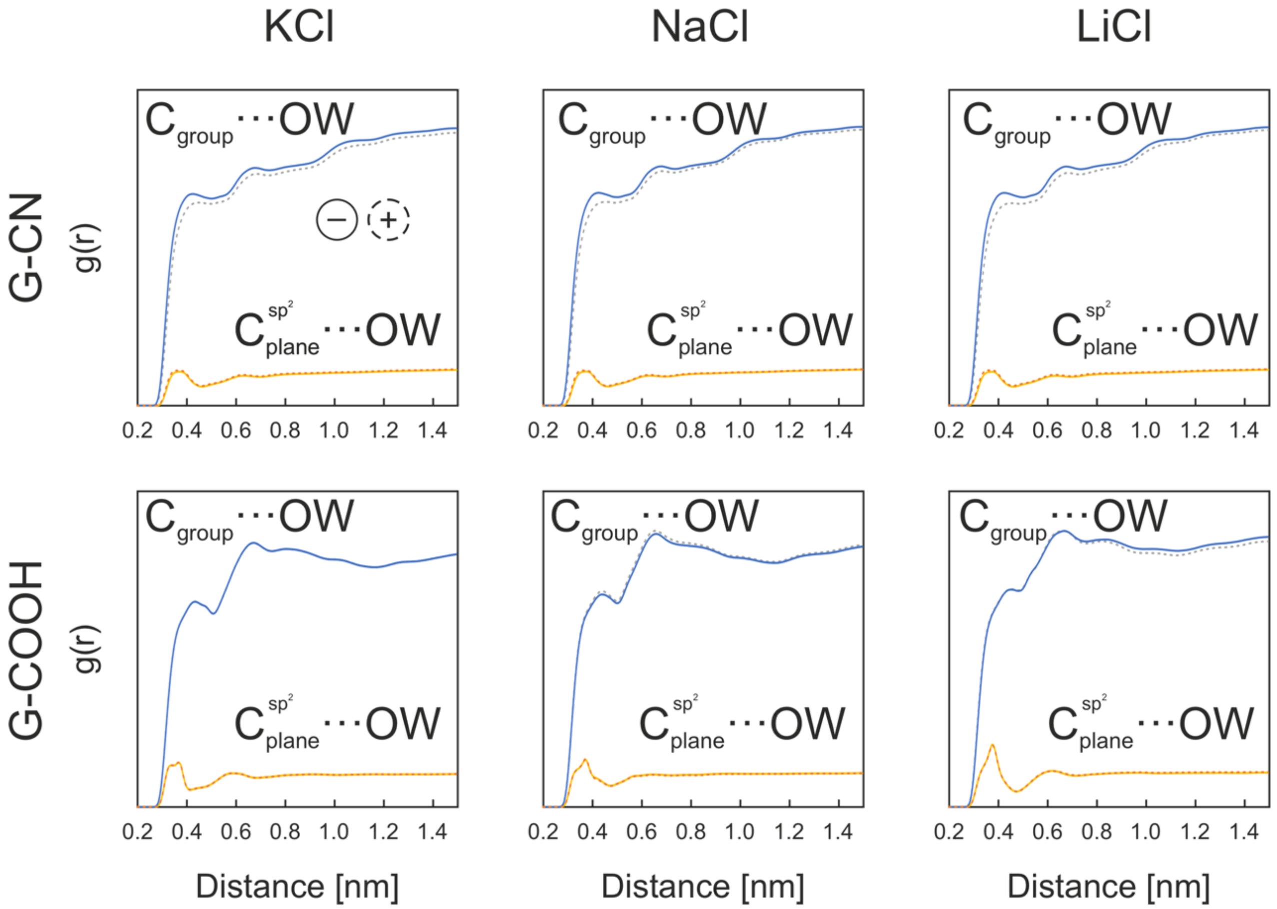

Figure S21. Radial distribution functions between the carbon in functional group/plane and the water oxygen for individual alkali-metal chloride electrolytes for G-CN and G-COOH. The positive charged electrode is shown using the dashed line.

References

[1] D. Zaoralová, V. Hrubý, V. Šedajová, R. Mach, V. Kupka, J. Ugolotti, A. Bakandritsos, M. Medved', M. Otyepka, *ACS Sustainable Chem. Eng.* **2020**, *8*, 4764.

[2] X. Wang, C. G. Bazuin, C. Pellerin, *Vibrational Spectroscopy* **2014**, *71*, 18.

[3] D. D. Chronopoulos, A. Bakandritsos, M. Pykal, R. Zbořil, M. Otyepka, *Applied Materials Today* **2017**, *9*, 60.

[4] J.-B. Wu, M.-L. Lin, X. Cong, H.-N. Liu, P.-H. Tan, *Chem. Soc. Rev.* **2018**, *47*, 1822.

[5] V. Šedajová, A. Bakandritsos, P. Błoński, M. Medveď, R. Langer, D. Zaoralová, J. Ugolotti, J. Dzíbelová, P. Jakubec, V. Kupka, M. Otyepka, *Energy Environ. Sci.* **2022**, *15*, 740.
